# Aseismic transient during the 2010–2014 seismic swarm: evidence for longer recurrence of M ≥ 6.5 earthquakes in the Pollino gap (Southern Italy)?

**DOI:** 10.1038/s41598-017-00649-z

**Published:** 2017-04-12

**Authors:** Daniele Cheloni, Nicola D’Agostino, Giulio Selvaggi, Antonio Avallone, Gianfranco Fornaro, Roberta Giuliani, Diego Reale, Eugenio Sansosti, Pietro Tizzani

**Affiliations:** 1 Istituto Nazionale di Geofisica e Vulcanologia (INGV), Centro Nazionale Terremoti, via di Vigna Murata 605, 00143 Rome, Italy; 2 Consiglio Nazionale delle Ricerche (CNR), Istituto per il Rilevamento Elettromagnetico dell’Ambiente, via Diocleziano 328, 80124 Naples, Italy; 3 Dipartimento della Protezione Civile (DPC), Ufficio Rischio Sismico e Vulcanico, via Vitorchiano, 2, 00189 Rome, Italy

## Abstract

In actively deforming regions, crustal deformation is accommodated by earthquakes and through a variety of transient aseismic phenomena. Here, we study the 2010–2014 Pollino (Southern Italy) swarm sequence (main shock *M*_*W*_ 5.1) located within the Pollino seismic gap, by analysing the surface deformation derived from Global Positioning System and Synthetic Aperture Radar data. Inversions of geodetic time series show that a transient slip, with the same mechanism of the main shock, started about 3–4 months before the main shock and lasted almost one year, evolving through time with acceleration phases that correlate with the rate of seismicity. The moment released by the transient slip is equivalent to *M*_*W*_ 5.5, significantly larger than the seismic moment release revealing therefore that a significant fraction of the overall deformation is released aseismically. Our findings suggest that crustal deformation in the Pollino gap is accommodated by infrequent “large” earthquakes (*M*_*W*_ ≥ 6.5) and by aseismic episodes releasing a significant fraction of the accrued strain. Lower strain rates, relative to the adjacent Southern Apennines, and a mixed seismic/aseismic strain release are in favour of a longer recurrence for large magnitude earthquakes in the Pollino gap.

## Introduction

The way in which a fault releases the accumulated tectonic strain during the interseismic period is a central question in seismotectonics and it has important implications in terms of crustal rheology and earthquake source mechanics. Moreover, the evaluation and the interpretation of the balance between seismic and geodetic release have key practical implications for seismic hazard assessment. In recent years, the increasing availability of geodetic data such as continuous Global Positioning System (GPS) observations and short repeat-time Synthetic Aperture Radar (SAR) images in combination with seismological data, have greatly increased our capability to discover transient aseismic slow slip episodes of different extent, duration and temporal evolution. These episodes are frequently accompanied by a variety of seismic phenomena^1–8^ that, in some cases seem to be the primary way in which the accrued tectonic stresses are released. While examples of post-seismic (afterslip) transients triggered by the rapid stress release in a main shock are well documented in several tectonic contexts^8–10^, most of the well-constrained sources of spontaneous aseismic slow slip come from subduction zones^2–6, 11^, such as Japan, Cascadia, Alaska, Mexico, New Zealand and Costa Rica and are referred to as slow slip events. Similar quasi-static slips have also been observed along the creeping section of the San Andreas Fault in California^12^, the Kilauea volcano in the Hawaii^13^ and, more recently, along the North Anatolian Fault in Turkey^7^ as well. Other kind of transient aseismic slow slip events have been hypothesized in association with earthquake swarms along active transform plate boundaries^14–16^ and in volcano active regions^13, 17, 18^. Although swarms are commonly related to high pore fluid pressure in the crust^19, 20^, other studies^21^ have instead suggested that aseismic processes may be a general feature of seismic swarms; however, very little information about surface deformation is usually available. Only in recent years, thanks to the development of space geodetic techniques, high spatial- and temporal- resolution surface measurements have been used to better understand the faulting behaviour of earthquake swarms, such as rupture details associated with the major individual events, as well as larger-scale deformation patterns of the whole swarm seismic process^6, 15, 16, 22, 23^.

**Figure 1:**
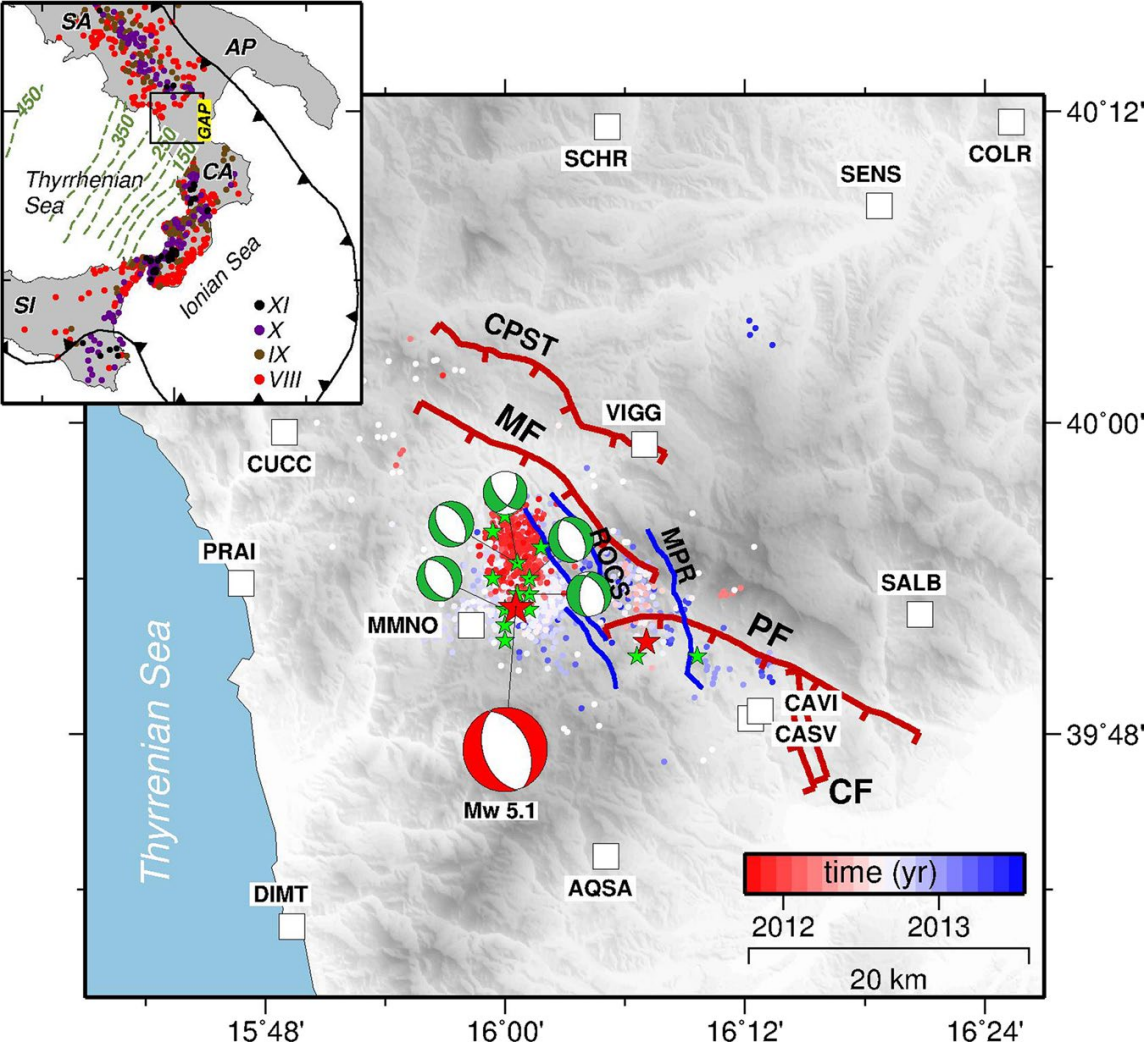
Tectonic setting of the Pollino swarm seismic sequence. The dots show the seismicity for the 2010–2014 Pollino earthquake swarm sequence, colour-coded by their time of occurrence^32^. The epicentre of the largest shock (*M*_*W*_ 5.1) is shown as a red star. The green stars indicate the location of the *M*_*W*_ > 3.5 events^27^. The mechanisms of these events are taken from time domain moment tensor (TDMT) catalogue (red and green beach-balls, http://cnt.rm.ingv.it/tdmt) and from Passarelli *et al*.^27^. White squares indicate the locations of the continuous GPS sites used in this work. The red lines represent the major mapped W-SW- dipping normal tectonic structures^34, 36, 37^: MF, PF, CF and CPST stand for Mercure, Pollino, Castrovillari and Castello Seluci-Timpa della Manca fault, respectively. The blue lines are the new recently identified active faults after Brozzetti *et al*.^37^: ROCS stands for the Rotonda-Campotenese normal fault system and MPR for the Morano Calabro-Piano di Ruggio fault. The inset shows the tectonic setting of Southern Italy and the historical macroseismic intensities^52^. Deep and intermediate seismicity in the Wadati-Benioff zone beneath the Tyrrhenian Sea, shown as contours of the subducted slab labeled in kilometers. The black lines with triangles represent the Plio-Pleistocene subduction front. The box encloses the main figure. AP = Apulia; SA = Southern Apennines; CA = Calabria; SI = Sicily. The map was created by using Generic Mapping Tools software (GMT v4.5.14; http://gmt.soest.hawaii.edu/)^53^.

Here, we use geodetic and seismological observations to document a transient aseismic slow slip event occurring during a years-long earthquake swarm that significantly contributed to the total release of the seismic moment. We inverted the 3-components GPS time series and the line-of-sight (LOS) displacements derived by processing data acquired by the COSMO-SkyMed (CSK) SAR satellite constellation with multi-temporal Differential SAR Interferometry (DInSAR) techniques, to estimate the temporal evolution of the transient slow slip event that accompanied the swarm sequence. This transient took place in the so-called Pollino seismic gap, Southern Italy, affected by an intense swarm sequence that started in October 2010 and lasted until the beginning of 2014^24–27^. The Pollino range is located between the end of the Southern Apennines extensional domain and the Calabrian arc^28–30^. It represents a well-known seismic gap in Italy^31^ due to a lack of local high macroseismic intensities, a feature which is usually indicative of a “large” earthquake (*M*_*W*_ ≥ 6.5) occurring on a nearby active fault (Fig. 1).

The 2010–2014 Pollino swarm sequence comprises more than 6000 events, as recorded by the Italian seismic network^32^, and provides an unprecedented opportunity to characterize a normal faulting earthquake swarm using both seismic and geodetic observations. The sequence started at the end of 2010 following decades of seismic quiescence^33^ and lasted until the beginning of 2014. The swarm contained a *M*_*W*_ 5.1 main shock which occurred on 25 October 2012, and which represents one of the largest earthquakes seismically recorded in this area (the only other significant event that occurred during the instrumental era is a *M*_*W*_ 5.6 earthquake, took place in 1998 to the north of the Pollino range). Most of the 2010–2014 swarm activity occurred in the hanging-wall of the large NW-SE striking normal fault zone that bounds the Pollino range^34, 35^. The 3-D patterns of the relocated hypocenters^24–26^ of the larger and more intense western cluster together with focal mechanisms of the largest events^26, 27^ (>3.5), consistently reveal a N-NW-striking and W-SW-dipping normal fault zone with centroid depths between 5 and 10 km. Although the SW-dipping focal plane is in agreement with the structures that represent the most common faulting style^34–37^, Totaro *et al*.^26^ and Brozzetti *et al*.^37^ demonstrated that the hypocentral distribution was not compatible with previous maps of known active faults^34–36^. In particular, in a recent study, Brozzetti *et al*.^37^ reconstructed a previously unidentified Late Quaternary extensional fault system, suggesting that a suitable source for the 25 October 2012 earthquake could be the previously unknown W-SW-dipping Rotonda-Campotenese fault system (ROCS), while the Morano Calabro-Piano di Ruggio (MPR) fault system could have controlled the eastern cluster of seismicity. The temporal and spatial behaviour of the recorded seismicity, as described by Passarelli *et al*.^27^, is consistent with the general characteristics of swarm-like seismicity^38, 39^. In particular, the sequence has affected a much larger crustal volume than expected according to the largest recorded event (*M*_*W*_ 5.1), with a significant enlargement of the focal area during the sequence (Fig. 1). The relationship between the spatial dimensions, the seismic moment released by the swarm sequence and ETAS (Epidemic Type Aftershock Sequence)^40^ modelling of the seismicity, has led to hypothesize that a transient forcing was acting during the Pollino swarm^27^. However, while being critical to reduce uncertainties in seismic hazard assessment due to seismic swarms, the nature of this transient forcing (which may range from aseismic creeping to diffusion of high pore pressure pulses, or even to fluid migration within the crust) has not been unravelled by previous studies^27^, lacking the observation capabilities to verify whether a transient aseismic slip episode actually accompanied the swarm.

Recent studies^41^ reviewed the historical seismicity in the Pollino range with magnitude comparable with the 25 October 2012 *M*_*W*_ 5.1 normal faulting event (depth 5 km), pointing out at least two similar events (i.e. in 1693 and 1708) occurred during a year-long seismic sequence, suggestive of a distinctive character for the seismicity in the Pollino area. Paleoseismological trenching studies^42, 43^ on the normal faults at the southern border of the Pollino range suggest the occurrence of at least four *M*_*W*_ ≥ 6.5 events within the last 10,000 yr. Recent preliminary estimates of tectonic loading show that the Pollino range is actively deforming, probably slower than the Southern Apennines (the latter characterized by deformation rates up to 2–2.5 mm/yr^44, 45^), with geodetic directions of active deformation consistent with the focal mechanisms of the largest events of the 2010–2014 Pollino swarm^27^.

Our combined analysis of the spatial-temporal evolution of seismicity and surface deformation associated with the Pollino seismic swarm sequence shows that an aseismic transient slip initiated several months before the main shock. Our results, indicate that an aseismic fault slip may have been the primary driving process of the Pollino swarm, suggesting that crustal deformation in the Pollino range may be characterized by aseismic slip episodes that release a significant fraction of the accrued strain, ultimately increasing the recurrence of surface-rupturing seismic events (*M*_*W*_ ≥ 6.5).

## Results

**Figure 2:**
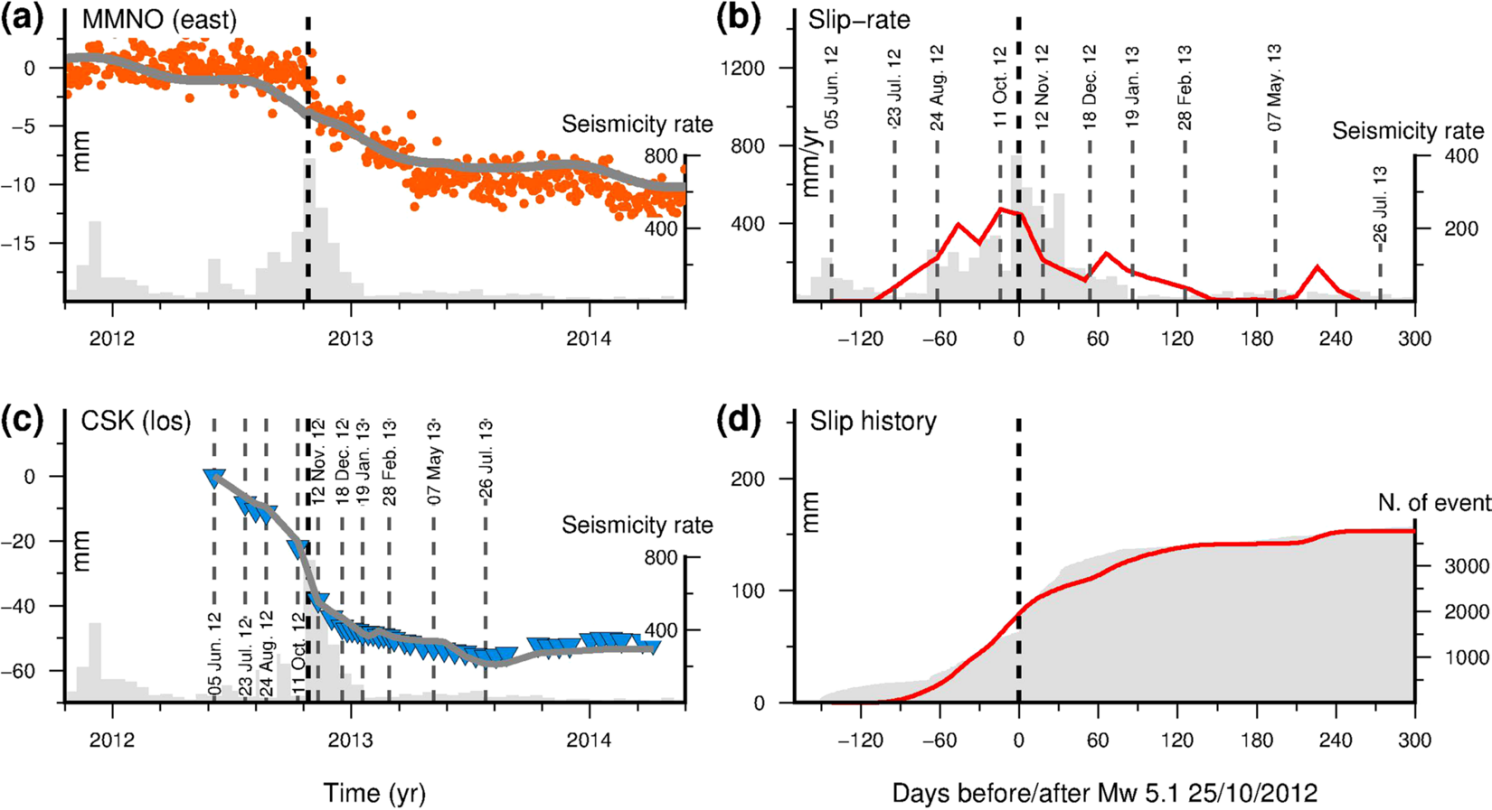
Geodetic time series, aseismic slip and seismicity rate. (**a**) Orange dots indicate the E-W (longitude) daily displacement recorded at GPS site MMNO in the Apulia (Ap) reference frame. The grey line is the prediction from the best-fit time dependent model of the aseismic transient slip event. The black dashed line indicates the 25 October 2012 *M*_*W*_ 5.1 main event. Histogram bars in panels (a) and (c) show the number of seismic events in 16-day intervals, while in panel (b) in 8-day intervals. The complete set of the GPS time series are in the Supplementary Material. (**b**) Best-fit aseismic slip rate estimated from the time dependent inversion (red line). Labelled dashed vertical lines indicated selected CSK acquisition epochs. (**c**) Blue triangles indicate the CSK light-of-sight (LOS) displacement observed in the area of maximum deformation (i.e., between MMNO and VIGG sites), while grey line is the prediction from the best-fit model. The transient event is clearly visible between July 2012 and the first half of 2013. (**d**) Evolution of cumulative aseismic slip (red line) and cumulative number of seismic events (grey area). The figures were created by using Generic Mapping Tools software (GMT v4.5.14; http://gmt.soest.hawaii.edu/)^53^.

**Figure 3:**
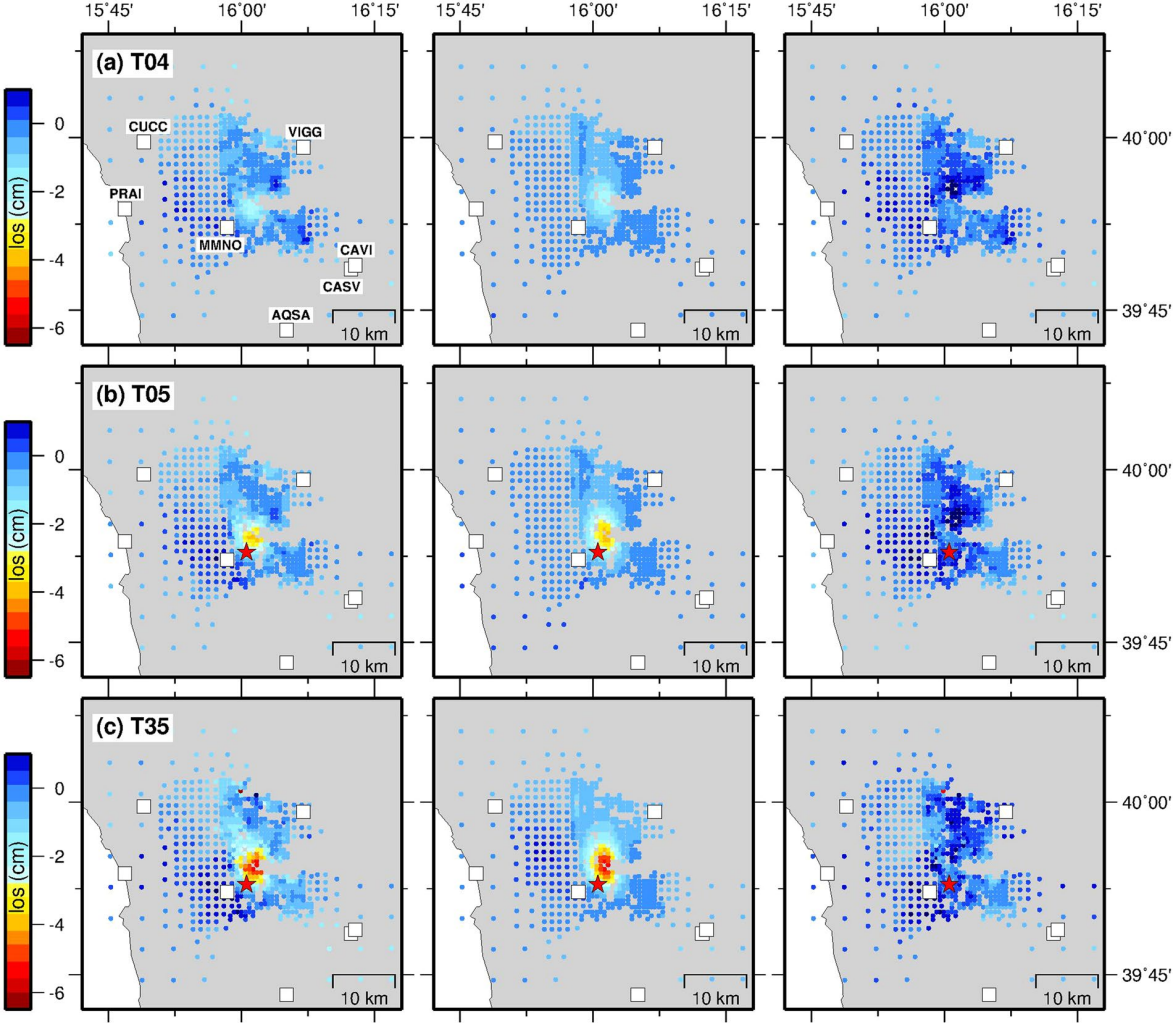
CSK DInSAR data around the Pollino range. Data (left panels), model (middle panels) and residuals (right panels) sampled points from CSK time series showing the displacement field as a function of time: (**a**) between 5 June-11 October 2012 (T04), (**b**) between 11 October-12 November 2012 (T05), and (**c**) between 12 November 2012–2 March 2014 (T35). Negative changes represent increase in radar LOS. The red star indicates the 25 October 2012 *M*_*W*_ 5.1 event. Details of interferometric pairs are shown in Supplementary Fig. S3. The complete set of the 36 epochs CSK displacement time series used in the inversion are fully described in the Supplementary Material. The maps were created by using Generic Mapping Tools software (GMT v4.5.14; http://gmt.soest.hawaii.edu/)^53^.

Geodetic measurements proved to be a crucial data set for understanding the 2010–2014 Pollino swarm sequence. In fact, both GPS and DInSAR time series clearly show a transient displacement starting before the 25 October 2012 *M*_*W*_ 5.1 main shock (Figs 2a and c). In particular, a surface displacement, mainly in the E-W direction, lasting several months from July 2012 to mid-2013, is well seen at the continuous GPS site MMNO, with a cumulative displacement up to ~10 mm in the west direction (Fig. 2a). On the contrary, the GPS daily time series and the GPS high-rate solutions (Supplementary Fig. S2) only show subtle coseismic offsets (<1–2 mm) associated with the main shock event. A similar signal is also present in nearby GPS stations (Supplementary Fig. S6a-l) with a smaller amplitude depending on the relative distance from the swarm. The transient displacement is even more clearly visible in the CSK time series measurements (Fig. 2c), which are also available in the area of maximum deformation (Fig. 3) where, unfortunately no GPS stations were operating. In this case, the cumulative displacement reaches ~60 mm in LOS direction. The comparison of the CSK time series with the independent GPS data at the MMNO station (Supplementary Fig. S5) demonstrates the consistency between the different data sets at the level of a fraction of a centimetre.

Our geodetic data highlight that surface deformation started in June 2012 and evolved until mid-2013, with alternating phases of acceleration and deceleration that correlate with the seismicity rate (Fig. 2). In particular, between June and October 2012 DInSAR displacement measurements (Fig. 3) reveal a cumulative LOS deformation of about 20 mm in the epicentral area before the occurrence of the main shock. Significant surface deformation is observed between 11 October and 12 November 2012 (interval containing the *M*_*W*_ 5.1 event), when the LOS cumulative displacement field reached a value of about 40 mm (Fig. 3, second row). In the following eight months, between 12 November 2012 and 26 July 2013, after about 1 year from the start of the detected transient surface deformation, the LOS displacement gradually achieved the final cumulative value of ~60 mm (the complete set of LOS displacement fields are shown in the Supplementary Fig. 7a–g).

**Figure 4:**
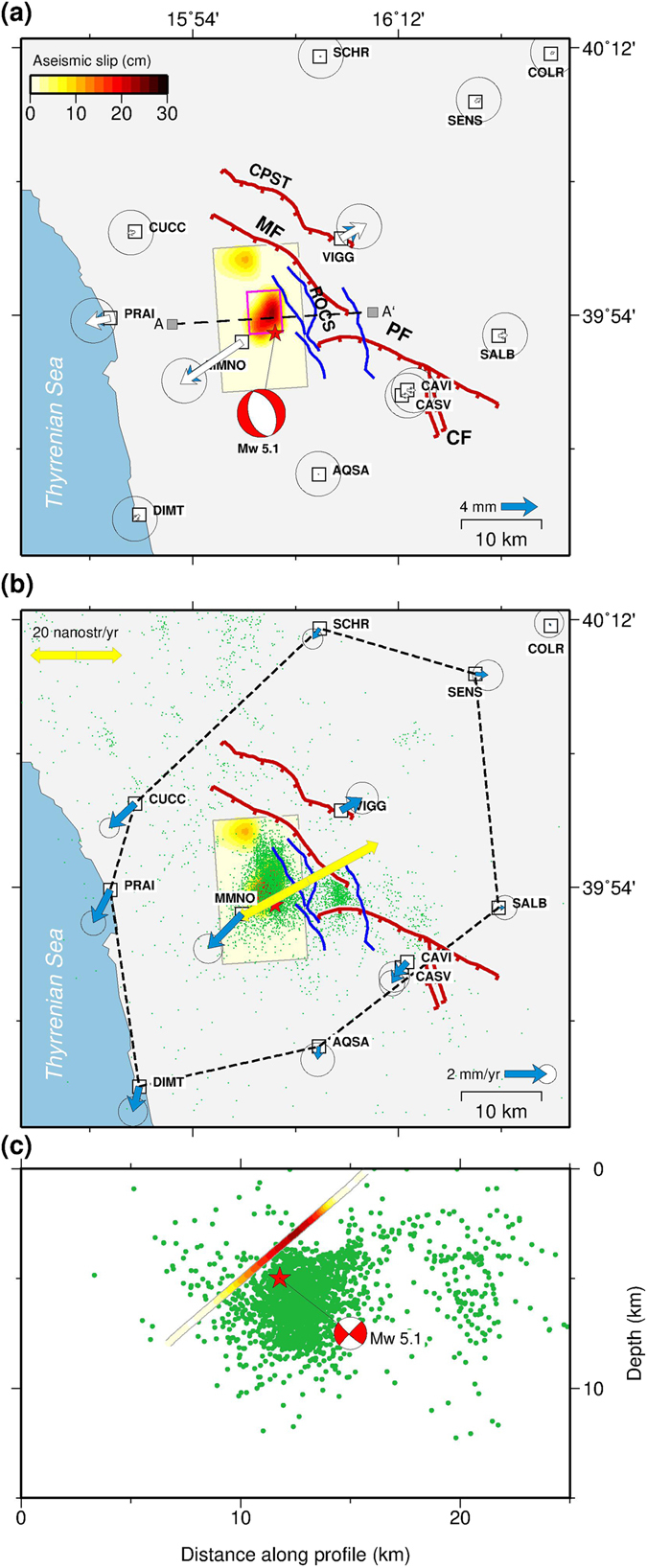
Surface deformation during the transient aseismic event and interseismic velocity field. (**a**) Observed (blue arrows) and predicted (white arrows) cumulative horizontal displacements from the aseismic model. The purple box represents the best-fit uniform slip aseismic fault plane, while the colour scale is the aseismic slip distribution (in mm) of the total cumulative displacement computed on an extended fault plane discretized into smaller patches. (**b**) Best-fit interseismic horizontal velocity field in an Apulian (Ap) reference frame. The dashed lines enclose the polygon used for strain rate calculation, while the double sided arrows indicate the principal strain rates (${\dot{\varepsilon }}_{max}$ = 34 ± 7 × 10^−9^  yr^−1^). Green dots represent the relocated^54^ seismic events during the swarm. Traces of active faults as in Fig. 1. (**c**) Estimated aseismic slip distribution as a function of depth (symbols as in panel c). The hypocentre of the largest shock (*M*_*W*_ 5.1) is shown as a red star and the mechanism of this event is taken from time domain moment tensor (TDMT) catalogue. The maps were created by using Generic Mapping Tools software (GMT v4.5.14; http://gmt.soest.hawaii.edu/)^53^.

Joint inversion of the 3-D continuous GPS time series at 12 sites and 35 DInSAR cumulative LOS displacement measurements spanning the swarm sequence, indicates that the main area of transient aseismic slip took place at shallow depths (between 2–7 km) along a source model which appears to be consistent with the mechanisms of the coseismic fault plane of the 25 October 2012 *M*_*W*_ 5.1 main shock (Fig. 4a). The surface projection of our best-fit model seems not to be fully compatible with the major mapped active faults^34–37^ in the Pollino area (that is, the Mercure and Pollino faults, MF and PF in Fig. 4a). On the other hand, our solution is consistent with recent studies^26, 37^ that have shown two newly identified sub-parallel W-SW-dipping fault segments (ROCS in Fig. 4a) as the main causative source of the Pollino swarm sequence.

The maximum cumulative slip reaches about 250 mm (Fig. 4a) and the moment release is equivalent to a magnitude *M*_*W*_ 5.5 earthquake. The cumulative moment release through earthquakes (including the *M*_*W*_ 5.1 event) in the corresponding time period is equivalent to *M*_*W*_ 5.17, indicating that the deformation occurring during the Pollino swarm sequence was about 70% aseismic. Both GPS and CSK time series (Fig. 2) show that this aseismic transient slip evolved with time. In fact, we observed phases of acceleration and deceleration of the aseismic slip which correlate with increase and decrease of the seismicity rate, respectively (Fig. 2b). In particular, between June and August 2012, about 3–4 months before the occurrence of the *M*_*W*_ 5.1 main shock, the rate of seismicity increased faster than in the previous 2011–2012 bursts of activity^27^, which unfortunately were not covered by the geodetic measurements. The aseismic slip rate shows a synchronous increment in almost the same time period, culminating in a dramatic increase just before the 25 October 2012 *M*_*W*_ 5.1 earthquake (Fig. 2). About two months after the main shock both the seismicity and the aseismic slip suddenly dropped off. The 2013 activity marks a new phase of swarm activity, with a significant enlargement of the area affected by the seismicity^27^. In this period, the aseismic slip rate gradually decreased until May 2013.

Between May and July 2013 (that is, between 200–240 days after the occurrence of the main event of the sequence) we observe another small increment in the slip rate. This increment is synchronous with an enlargement of the crustal area affected by the seismicity and is required to accommodate the surface deformation observed in our geodetic time series. Additionally, statistical analysis (i.e. ETAS modeling^27^) of the swarm, suggests that the transient forcing process lasted throughout all the seismic sequence and not just during the acceleration phases observed by our geodetic data. This leads to the conclusion that the slow-slip event is the main driver of the whole seismic sequence since October 2010.

## Discussions

We suggest that a transient aseismic slow slip event started about 3–4 months before the occurrence of the main shock (*M*_*W*_ 5.1), and systematically accompanied the seismic sequence (at least in the time span covered by the geodetic observations). The start of the aseismic transient coincides with the fast increase of the seismicity rate detected by Passarelli *et al*.^27^ and ascribed to an aseismic transient forcing. The observed increasing and decreasing seismicity rate, were accompanied by the transient acceleration and deceleration of the aseismic slip respectively (Fig. 2). The surface deformation increased with time, reaching up to ~10 mm at MMNO GPS station and about 60 mm in the LOS at the end of the transient. Furthermore, the signal amplitude and the spatial extent of deforming area clearly increase with time (Fig. 3). Therefore, the LOS changes and GPS surface deformation across the Pollino range are observed not only during the *M*_*W*_ 5.1 event, but also before and after the main shock, thus demonstrating that aseismic slip occurred during the seismic swarm. Both seismic and aseismic moment release contributed to the total release of the tectonic strain accumulated during the interseismic phase. For this reason, the detection and estimation of the transient aseismic phenomena have significant implications for the evaluation of the fraction of tectonic loading released seismically which in turn has consequences in terms of seismic hazard. Furthermore, it is important to identify other possible faults in the Pollino seismic gap region that may have been brought closer to failure by the stress changes associated with the estimated aseismic transient slip episode. Apart from the obvious increased stress on the portion of the causative fault surrounding the aseismically slipped area, we find very little stress increase (about 0/0.2 bar) in the north-western tip of the southern PF fault (Supplementary Fig. S8). We find a general decrease in stress (−3.8/−0.8 bar) on the south-eastern half of both the MF and CPST fault planes, and a stress increase (up to 1.5 bar) on their north-western parts (Supplementary Fig. S8) which may represent a feature that should be considered in future hazard assessment (a complete description of the static stress changes calculation is given in the Supplementary Material).

The observed aseismic transient fault slip implies that the Pollino range faults should have accumulated interseismic elastic strain before the swarm sequence. Figure 4b shows the interseismic velocities corrected for the transient displacements occurred during the Pollino swarm. Our estimate of secular tectonic loading is ~1.7 mm/yr (Fig. 4b), thus showing a significant southward decrease of active extension from the Southern Apennines (extension ~2.5 mm/yr^44, 45^) to the Pollino range. However, the definition of the interseismic behaviour for the Pollino range active faults is challenging due to the coverage of the geodetic network in the region which poorly resolves the main deformation mechanism active on the faults in the Pollino area. A long-lasting (at least one decade) centimetre scale creeping behaviour has been suggested by Sabadini *et al*.^46^ on the basis of non-continuous DInSAR and GPS data. However, discrepancies between the proposed rates and the regional tectonic loading and the lack of surface expression along the trace of the involved faults does not appear to fully support this hypothesis. On the other hand, if we assume that the Pollino range fault systems behave the same way as those involved during the seismic swarm, then the creeping reported on the southern fault system by Sabadini *et al*.^46^ could be interpreted as the superimposition of several episodic aseismic slip transients.

The behaviour observed during the Pollino swarm sequence suggests that the seismically-radiating faults (velocity-weakening patches) may be heterogeneously distributed in a spotty style, while velocity-strengthening zones could be more widely distributed on the fault. The *M*_*W*_ 5.1 main shock has nucleated on a velocity-weakening patch, although our interseismic velocity field cannot resolve the accurate geometry of this structure. Paleoseismological data^41, 42^ from the southern fault segments of the Pollino fault system suggests that the dimensions of velocity-weakening patches are not limited to *M*_*W*_ ≈ 5 events, but may reach sizes capable of generating a surface-rupturing event (*M*_*W*_ ≥ 6.5).

**Figure 5:**
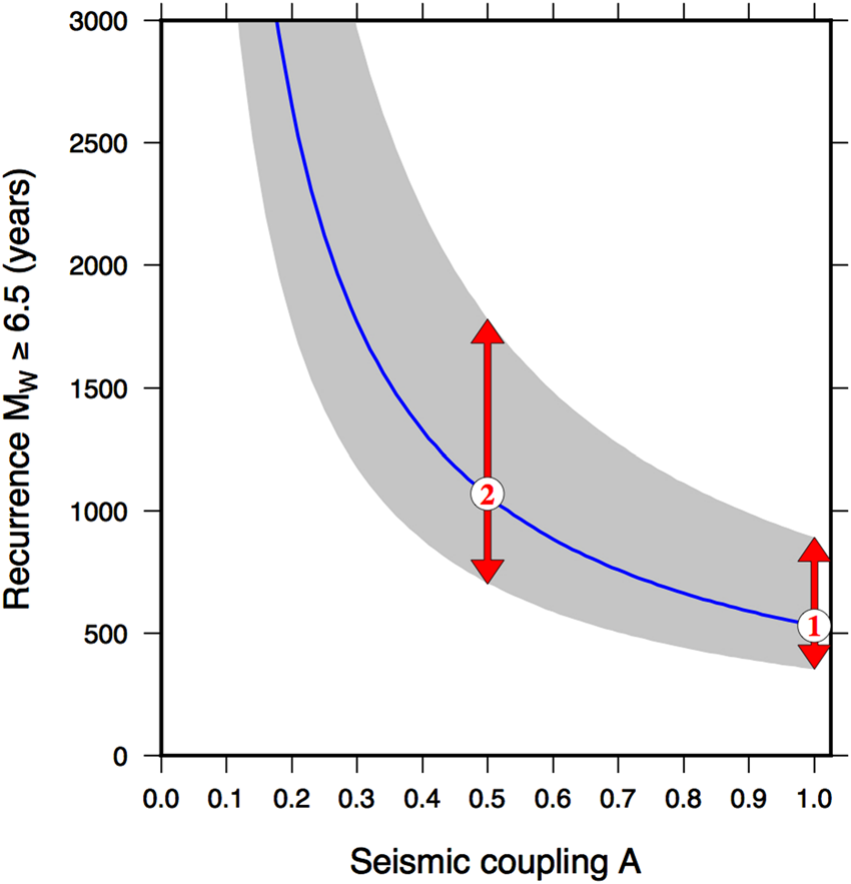
Recurrence of *M*_*W*_ ≥ 6.5 events derived from the interseismic geodetic strain rate. The blue line shows the recurrence of *M*_*W*_ ≥ 6.5 events as a function of seismic coupling fraction. The grey area includes the ±1-sigma uncertainty. The recurrence estimates have been calculated using ${\dot{\varepsilon }}_{max}$ = 34 ± 7 × 10^−9^ yr^−1^ and *H* = 10 ± 2.5 km. Full seismic coupling (*c* = 1.0, case 1) predicts a *M*_*W*_ ≥ 6.5 event every 350–890 years. Allowing half of the tectonic loading to be released aseismically (*c* = 0.5, case 2), doubles the recurrence to 700–1780 years and increase the probability of not observing large macroseismic intensities in the Pollino seismic gap area. The figure was created by using Generic Mapping Tools software (GMT v4.5.14; http://gmt.soest.hawaii.edu/)^53^.

One important consequence of the transient aseismic slip is that the associated expected rate of large earthquakes is lower than the one envisaged from a full velocity-weakening behaviour and a full seismic release. We calculate the recurrence of *M*_*W*_ ≥ 6.5 events predicted by the observed interseismic strain rate (corrected for the effect of the aseismic transient, Fig. 4b) by accounting for the effect of different fraction of the aseismic deformation (see Methods). Figure 5 shows that complete seismic release of the tectonic loading (case 1 in Fig. 5) requires a *M*_*W*_ ≥ 6.5 event every 350–890 years. This value is slightly lower than, but similar to, the recurrence of *M*_*W*_ ≥ 6.5 events in the Central-Southern Apennines (240–600 years), where the spatial distribution of large macroseismic intensities in the last 1000 years does not show significant gaps^47^. Halving the seismic coupling (case 2 in Fig. 5) doubles the recurrence of *M*_*W*_ ≥ 6.5 events and increases the probability of not observing large macroseismic intensities in the historical catalogue. Thus, the combination of lower strain rates relative to the adjacent Southern Apennines, and a mixed seismic/aseismic strain release may be a possible scenario capable of increasing the recurrence time of large magnitude events in the Pollino seismic gap.

## Methods

### GPS data and processing

Surface displacements have been recorded by 12 permanent Global Positioning System (GPS) stations managed by different public and private institutions (Fig. 1). GPS data were processed using the Jet Propulsion Laboratory (JPL) GIPSY-OASIS II software. A complete description of the processing details and strategies are given in the Supplementary Material. Visual inspections of the GPS time series (Supplementary Fig. S6a–l) only show subtle coseismic offsets (<1–2 mm) related to the 25 October 2012 *M*_*W*_ 5.1 main event of the swarm sequence. On the contrary, the two GPS sites located closest the source (MMNO and VIGG) are likely affected by significant (>5 mm) transient deformation in the E-W component, especially following the *M*_*W*_ 5.1 event (Fig. 2). A similar signal is also present in the other nearby stations (Supplementary Fig. S6a–l).

### High-rate GPS analysis

A high-rate analysis of GPS data at the closest stations (MMNO and VIGG) is performed with the strategy described in the Supplementary Material. This analysis results in 30 sec-sampled time series covering a 1.5-hour time interval spanning the *M*_*W*_ 5.1 earthquake (Supplementary Fig. S2). The uncertainties of these time series are 0.45, 0.39 and 1.09 cm for the North, East and Vertical components, respectively. The signals produced by the *M*_*W*_ 5.1 event are within the uncertainties of the high-rate GPS solutions and no clear offsets seem to occur during the largest earthquake of the swarm sequence.

### DInSAR data and processing

We used Synthetic Aperture Radar (SAR) data acquired by the COSMO-SkyMed (CSK) constellation, composed of four satellites and operated by the Italian Space Agency. A temporally dense data set was available thanks to a specific acquisition planning conveniently managed during the seismic crisis. Images were acquired in ascending orbits (side-looking angle of about 30° off the vertical) in the stripmap (HIMAGE) mode with 3 m by 3 m spatial resolution. A data set of 39 stripmap images was available: the time interval covered by the acquisitions starts on 5 June 2012 and includes almost two years of surface deformation up to 8 April 2014. Acquisition parameters in terms of temporal and spatial baselines with respect to the reference master image acquired on 23 May 2013, are listed in Supplementary Table T3 and are shown in Supplementary Fig. S3. Data have been processed with a two-scale approach, at low resolution (small scale) and high resolution (large scale). A complete description of the processing details and strategies is given in the Supplementary Material.

Figure 2 and Supplementary Figs S5 and S7a–g show the temporal evolution of the line-of-sight (LOS) displacement derived from the time series analysis in the area of maximum deformation (i.e., between MMNO and VIGG stations). Two first clear (≥1 cm) LOS displacements occurred before the *M*_*W*_ 5.1 earthquake. In particular, between the first two acquisition dates of 5 June and 23 July 2012, and between 24 August and 11 October 2012, when the surface deformation reached a cumulative LOS value of >2 cm. Between 11 October and 12 November 2012, that is time interval spanning the 25 October 2012 *M*_*W*_ 5.1 earthquake, we observed another significant (>2 cm) phase of rapid surface deformation. Finally, in the following months the surface deformation continued until the middle of 2013, but at a slower rate, reaching a total cumulative LOS displacement >6 cm.

We analysed the GPS and DInSAR time series by projecting the GPS positions along the CSK LOS and comparing the resulting values with the CSK displacements averaged within 150 meters from the GPS monument (Supplementary Fig. S5). In particular, the comparison of the CSK time series with the independent GPS time series of MMNO station demonstrates the consistency between the different data sets of measurements at the level of a fraction of a centimetre.

### Time dependent inversion

We inverted the 3-D GPS time series and DInSAR displacement fields to simultaneously estimate the coseismic displacement related to the *M*_*W*_ 5.1 earthquake source and the aseismic transient slow slip event. To emphasize the extensional deformation across the Southern Apennines, the time series are shown (Supplementary Figs S1 and S6) in a reference frame defined by minimizing the horizontal velocities of the stations in the Apulian block^44^. The inversions were performed with TDEFNODE^48^. Because of the limited number of GPS stations and of the simple concentric deformation pattern observed in the DInSAR displacement fields, we assume a uniform slip on the rupture plane.

In particular, we modelled the *M*_*W*_ 5.1 event as a 4 km by 4 km square dislocation with a uniform slip of 10 cm and we fixed the fault strike, dip, rake and hypocentral depth (164°/47°/−84°/5 km) according to the focal mechanism solution of the TDMT catalogue (http://cnt.rm.ingv.it/tdmt). The synthetic offsets produced by this source are in agreement with the small static coseismic offsets observed both in the daily and in the high-rate 30-sec sampled GPS solutions (Fig. S2). The aseismic transient slow slip event was modelled as a planar uniform slip source with time dependence set as a series of overlapping triangles. We inverted for the dimensions, positions and strike, dip and rake of the fault plane. In addition, for a slow slip event, the free parameters for the time history are the origin time, *T*_*0*_, and the triangle amplitudes, *A*_*i*_ (where *i* is the progressive number of the triangle in the time function). The rise time of the triangle is fixed at 16 days. To test variable slip on the fault plane, we also computed the slip distribution of the total cumulative displacement (Fig. 4a). A full explanation of the inversion scheme and tests are given in the Supplementary Material.

### Seismic moment accumulation and seismic potential

To estimate the rate of seismic release and the effect of aseismic deformation, we computed the rate of seismic moment accumulation from the geodetic strain rate in the polygon in Fig. 4b using a scalar version of the Kostrov's formula^49^:

1 $${\dot{M}}_{geod}=2\mu AT{\dot{\varepsilon }}_{max}$$

where ${\dot{\varepsilon }}_{max}$ is the largest absolute eigenvalue of the strain rate tensor, *A* is the considered area, *T* is the seismogenic thickness and *μ* is the rigidity modulus (3.3 × 10^10^ Pa). The rate of seismic release is evaluated under the assumption that the seismic moment is distributed across earthquakes obeying the Gutenberg-Richter relation between magnitude and frequency truncated to a maximum moment earthquake^50^:

2 $$\dot{N}({M}_{0})=\alpha {M}_{0}^{-\beta }[1-H({M}_{0}-{M}_{0}^{max})]$$

where $\dot{N}$ is the rate of events having moment greater than or equal to *M*_*0*_, ${M}_{0}^{max}$ is the moment of the maximum magnitude event*, H* is the Heaviside function and *β* = 2/3 (equivalent to assuming *b* = 1 in the Gutenberg-Richter relation). The rate of total moment release is refs 50, 51:

3 $${\dot{M}}_{0}^{tot}=\frac{\alpha \beta {M}_{0}^{max(1-\beta )}}{1-\beta }$$

Equation (3) can be reformulated and *α* inserted in (2) thus leading to:

4 $$\dot{N}({M}_{0})=c\frac{{\dot{M}}_{0}^{tot}(1-\beta )}{\beta {M}_{0}^{max(1-\beta )}}{M}_{0}^{-\beta }$$

We assume the magnitude of the maximum event *M*_*max*_ = 7.0 (similar to the estimated maximum magnitude of the largest events observed in the Apennines^31^). We also introduced the value *c* to account for a variable fraction (between 0 and 1) of seismically released ${\dot{M}}_{0}^{tot}$ .

## Electronic supplementary material

Supplementary Information
