# Supplementary material for: Aseismic transient during the 2010–2014 seismic swarm: evidence for longer recurrence of M ≥ 6.5 earthquakes in the Pollino gap (Southern Italy)?
Source: Sci Rep. 2017 Apr 12;7:576. doi: 10.1038/s41598-017-00649-z (PMC5429810; doi:10.1038/s41598-017-00649-z)
Supplement: Supplementary file 1 — Supplementary Information [file 41598_2017_649_MOESM1_ESM.pdf]

Supplementary Information

Aseismic transient during the 2010-2014 seismic swarm: evidence for longer recurrence of M≥6.5 earthquakes in the Pollino gap (Southern Italy)?

Daniele Cheloni[1,*], Nicola D'Agostino[1], Giulio Selvaggi[1], Antonio Avallone[1], Gianfranco Fornaro[2], Roberta Giuliani[3], Diego Reale[2], Eugenio Sansosti[2] & Pietro Tizzani[2]

[1]Istituto Nazionale di Geofisica e Vulcanologia (INGV), Centro Nazionale Terremoti, via di Vigna Murata 605, 00143, Rome, Italy
[2]Consiglio Nazionale delle Ricerche (CNR), Istituto per il Rilevamento Elettromagnetico dell'Ambiente, via Diocleziano 328, 80124, Naples, Italy
[3]Dipartimento della Protezione Civile (DPC), Ufficio Rischio Sismico e Vulcanico, via Vitorchiano, 2, 00189, Rome, Italy

*corresponding: daniele.cheloni@ingv.it

1. Supplementary Methods

GPS processing strategy

We used the daily rinex files from permanent GPS stations in Southern Italy, most of which belong to the Rete Integrata Nazionale GPS network (RING[1]; available at http://ring.gm.ingv.it). Site coordinates of stations from RING and additional networks can be found in Table S1. GPS data are reduced using the Jet Propulsion Laboratory (JPL) GIPSY-OASIS II software (ver. 6.3) in a Precise Point Positioning mode applied to ionospheric-free carrier phase and pseudorange data[2] and using JPL's final fiducial-free GPS orbit products. We apply the VMF1 grids tropospheric mapping function[3] and estimate tropospheric wet zenith delay and horizontal gradients as stochastic random-walk parameters every 5 min[4], to model tropospheric refractivity. We compute the ocean loading from the FES2004 tidal model coefficients provided by the Ocean Tide Loading Provider[5] (http://holt.oso.chalmers.se/loading) and apply it as a station motion model. Ambiguity resolution is applied using the wide lane and phase bias (WLPB) method[6]. In order to analyze and interpret station velocities relative to the Eurasia plate and to reduce the common mode signal, we updated the Eurasian terrestrial reference frame described in Métois *et al.*[7]. This frame is defined by 6 Cartesian coordinates and velocities for 132 stations selected by specific quality criteria. Our Eurasian frame is aligned in origin and scale with IGS08[8] and it is implemented to have no-net rotation with respect to the stable interior of the Eurasian plate, realized by a 32-station core subset. In order to emphasize the long-term, secular ~3mm/yr NE-SW directed active extension across the Apennines, crustal velocities (Fig. S1) and time series used for the inversion are rotated relative to the Apulian block (Table S2). GPS velocities and related uncertainties (Fig. S1) are obtained using the robust trend estimator MIDAS[9]. The MIDAS- estimated velocity is essentially the median of the distribution of values calculated using pairs of data in the time series separated by approximately 1 year, making it insensitive to seasonal variation and time series outliers.

MIDAS provides uncertainties based on the scaled median of absolute deviations of the residual dispersion. The uncertainties have been shown to be realistic and do not require further scaling[9].

The high-rate GPS analysis strategy

In addition to the standard GPS processing strategy, we performed also a high-rate analysis of the GPS data at the two closest stations, MMNO and VIGG (Fig. S2). The high-rate GPS analysis strategy was performed by using the GIPSY-OASIS II software (ver. 6.3) released by Jet Propulsion Laboratory (JPL, http://gipsy-oasis.jpl.nasa.gov) and the JPLs final fiducial GPS orbits and high-rate (30 sec) clocks products[6]. The analysis strategy performed in this study was mainly based on three steps: first, using the Global Pressure and Temperature 2 (GPT2) troposphere refractivity function, a static solution with a 5-min sampling rate was performed to estimate the tropospheric dry and wet zenith delay and the horizontal gradients as stochastic random walk parameters; second, the dry troposphere contribution was extracted from the previous step and used, as well as the GPT2 mapping function, as input in the second static solution to improve the estimation of the wet troposphere zenith delay contribution; third, both the dry and wet troposphere contributions were used as input to estimate the station position epoch-by-epoch during the kinematic solution.
For all the steps, the precise point positioning method was applied to ionosphere-free carrier phase and pseudorange data[2] and a 2nd-order ionospheric correction was estimated by using the IRI ionospheric model[10,11]. Ocean loading was also computed from the FES2004 tidal model coefficients delivered by the Ocean Tide Loading Provider at Chalmers University[5] (http://holt.oso.chalmers.se/loading) to model station motion. Finally, absolute antenna calibration was used to improve the model of the GPS antennas and the ambiguity resolution was performed by using the wide-lane and phase bias (WLPB) method[6].

InSAR processing strategy

Interpretation of single interferograms encompassing the main shock generated during the seismic crisis was impaired by the presence of large atmospheric patterns that can be only handled by multi-temporal analysis carried out with Advanced Differential SAR Interferometry (A-DInSAR) approaches. Data have been processed with a two-scale (A-DInSAR) approach whereby a sequence of processing steps were carried out at low resolution (small scale) and high resolution (large scale), respectively. The low resolution processing involved a Small Baseline Subset (SBAS) DInSAR processing algorithm as described in Fornaro *et al.*[12], with a spatial resolution of about 50mx50m. This algorithm retrieved an estimation of the atmospheric propagation delay patterns as well as the deformation time series on a small spatial scale on almost the whole frame of 40Kmx40Km.
In all the CSK interferograms, topographic fringes were removed using the 1 arcsec resolution Shuttle Radar Topography Mission (SRTM) Digital Elevation Model. Moreover, a specific processing has been applied for the estimation and compensation on slowly temporally variable atmospheric components associated with seasonal stratified atmospheric components. The method, similar to that discussed in Fornaro *et al.*[13], applies a filter that removes components that would not be captured by the standard spatially low-pass and temporally high-pass filtering of atmospheric patterns.
The signal component retrieved at low resolution were used to calibrate the data for the subsequent higher resolution analysis. The latter was carried out via a tomographic based approach which has been demonstrated[14] to achieve improvements in the detection and estimation of topographic and motion parameters of persistent scatterers with respect to classical Persistent Scatterers Interferometry (PSI) methods.

The higher resolution processing benefited from a filtering method, named "Component extrAction and selEction SAR (CAESAR)" which has been recently proposed and patented. CAESAR achieves a favorable tradeoff between the final resolution and the spatial density of monitored pixels. It exploits a covariance based analysis to extract dominant components and achieve a dramatic increase in the detection of persistent scatterers by slightly sacrificing the spatial resolution of the final image. Further details on the CAESAR method are given in Fornaro *et al.*[15,16]: the final image has a resolution of 9mx9m, achieved averaging on a 3x3 box the full resolution CSK data with a very high density of monitored pixels, see Supplementary Fig. S4.
Before modeling, the DInSAR interferograms were downsampled using a resolution-based resampling algorithm[17,18]. The resampling technique permits us to reduce the number of data points from several millions to a set of about some hundreds of points, with the highest density of data points close to the source of deformations.

Time dependent inversion

Geodetic time series contain several signals, related both to the steady tectonic motions and to the transient motions. Time dependent inversions were performed with TDEFNODE[19]. In the TDEFNODE approach the parameters describing the steady motions are estimated simultaneously with the transients. The long term constant (or steady state) geodetic surface velocities are estimated by finding the best fit slope for each time series individually. As regard, the distribution of transient slip on the fault, in TDEFNODE it can be represented in a number of ways. The approach does allow considering both for uniform slip faults and distributed slip on discrete nodes, as well as simple functions describing the spatial distributions of slip on the fault. The slip rate, *s*, on the fault during an event is described by:

$$s(x,w,t) = A \cdot X(x) \cdot W(w) \cdot S(t)$$

where $X(x) \cdot W(w)$ is the spatial dependence, and *x* is along-strike position on the fault, *w* is the down-dip position, *t* is time and *A* is the amplitude. The time dependence, *S(t)*, of the slow slip event can be set to an impulse, a Gaussian function, a box-car function or a series of overlapping triangles as is done for earthquake time functions[20]. The surface displacement history is found by integrating *S(t)* over time and by applying the appropriate Green's functions. In TDEFNODE the inversions were done with a combination of grid search and simulated annealing. The quantity that is minimized is the sum of the reduced chi-square of the weighted misfit residuals plus any penalties (i.e., penalties that are assessed for exceeding parameter bounds). Parameter uncertainties are estimated by a linearization at the best fit parameter values, and for this reason are likely underestimated.
Here, we used a total of 87772 observations (63972 observations are the ENU component position of the GPS time series, while 23.800 observations are the LOS displacements) and a time history comprising overlapping triangles. In this case, the free parameters for the time history are T_0, the origin time, and the triangle amplitudes A_i (where *i* is the number of triangles in the time function and A_i is given in mm/yr). The aseismic slow slip transient event was represented by an origin time, 24 triangular time function elements, one slip amplitude and fault dimensions (length and width), fault strike, dip and rake and position. The resulting chi-square of the misfit residuals is 2.04, suggesting that the observation uncertainties are slightly underestimated or that a uniform slip fault model is a too simple model to explain the observed deformation.

Slip distribution inversion

The input data set for the inversion are the horizontal cumulative displacements observed at 12 GPS sites (Table T4) and the cumulative CSK interferogram (Fig. S7g). To test variable slip on the fault plane, the best fit fault was discretized into smaller patches of 1 x 1 km. We extended the fault from the surface to a depth of about 15 km in order to capture the entire area affected by the swarm sequence and the concentric displacement pattern revealed in the DInSAR interferograms, and to allow for down dip and along strike slip. We computed the Green's functions, which relate unit slip on individual fault patches to surface displacements at individual points using rectangular dislocations in an elastic, homogeneous, and isotropic half space[21]. The inversion was carried out using a bounded-values, weighted least squares inversion algorithm[22], to impose a nonnegative constraint on the estimated slip allowing unbounded values for InSAR orbital parameters (that is, offset and ramp), and using a Laplacian smoothing operator to avoid implausible slip distribution.

Stress transfer

It is well established[23-26] that stress transfer after a slip episode (both coseismic and aseismic slip) may load or discharge adjacent structures. It is therefore important to identify which other possible faults in the Pollino seismic gap area that may have been brought closer to failure by the stress changes following the mixed coseismic/aseismic transient slip episode occurred during the 2010-2014 Pollino swarm sequence. For this reason, we calculate the stress changes due to our best-fit model of the aseismic slip on the northern and southern normal faults in the region (ME, CPST and PF faults) using the Coulomb 3.1 code[26]. We assume a dip of 50° and a rake of -90° for each fault and subdivide them into 2X2 km patches (fault dimension about 20X16 km for each fault, friction coefficient 0.4). Obviously, the largest stress increases are on the patches of the causative fault surrounding the area that is modelled as being affected by the aseismic slip during the swarm sequence (Fig. S8). On the southern PF fault, we find little increased stresses (about 0/0.2 bar) only in the north-western tip of the fault plane, whereas on the northern MF and CPST faults do we find decreased stress (about -4/0.8 bar) on the two south-eastern halves of both the planes, and increased stresses (up to 1.5 bar) on their north-western parts. If we consider in the calculation the small patch of estimated aseismic slip from our variable slip model (located in the northern part of the extended fault plane), we find that stress is further decreased on the central parts of the northern faults. Since this feature might not be well resolved by our data, we suggest that the stress variation on the faults of the Pollino area is likely due only to the main patch of our estimated aseismic slip distribution.

References

1. Avallone, A. *et al.* The RING network: improvements to a GPS velocity field in the central Mediterranean. *Ann. Geophys.* **53**, 39-54 (2010).
2. Zumberge, J. F., Heflin, M. B., Jefferson, D. C., Watkins, M. M. & Webb, F. H. Precise point positioning for the efficient and robust analysis of GPS data from large networks. *J. Geophys. Res.* **102,** 5005-5017 (1997).
3. Boehm, J. Werl, B. & Schuh, H. Troposphere mapping functions for GPS and very long baseline interferometry from European Centre for Medium-Range Weather Forecasts operational analysis data. *J. Geophys. Res.* **111**, B02406, 10.1029/2005JB003629 (2006).
4. Bar-Sever, Y. E., Kroger, P. M. & Borjesson, J. A. Estimating horizontal gradients of tropospheric path delay with a single GPS receiver. *J. Geophys. Res.* **103**, 5019-5025 (1998).
5. Scherneck, H. A parametrized solid earth tide model and ocean tide loading effects for global geodetic baseline measurements. *Geophys. J. Int.* **106**, 677-694 (1991).
6. Bertiger, W. *et al.* Single receiver phase ambiguity resolution with GPS data. *J. Geod.* **84,** 327-337 (2010).
7. Métois, M. *et al.* Insights on continental collisional processes from GPS data: Dynamics of the peri-Adriatic belts. *J. Geophys. Res.* **120**, 8701-8719 (2015).

8. Rebischung, P. *et al.* IGS08: The IGS realization of ITRF2008. *GPS Sol.* **16**, 483-494 (2012).
9. Blewitt, G., Kreemer, C., Hammond, W. C. & Gazeaux, J. MIDAS robust trend estimator for accurate GPS station velocities without step detection. *J. Geophys. Res.* **121**, 2054-2068 (2016).
10. Bassiri, S. & Hajj, G. A. Higher-order ionospheric effects on the global positioning system observables and means of modelling them. *Manuscr. Geodaet.* **18,** 280-289 (1993).
11. Kedar, S., Hajj, G. A., Wilson, B. D. & Heflin, M. B. The effect of the second order GPS ionospheric correction on receiver positions. *Geophys. Res. Lett.* **30,** 1829 (2003).
12. Fornaro, G., Pauciullo, A. & Serafino, F. Deformation monitoring over large areas with multipass differential SAR interferometry: A new approach based on the use of spatial differences. *Int. J. Remote Sens.* **30**, 1455-1478 (2009).
13. Fornaro, G. *et al.* Assimilation of GPS derived atmospheric propagation delay in DInSAR data processing. *IEEE J. Sel. Topics Appl. Earth Observ. In Remote Sens.* **8**, 784-799 (2015a).
14. De Maio, A., Fornaro, G. & Pauciullo, A. Detection of Single Scatterers in Multidimensional SAR Imaging. *IEEE Trans. Geosci. Remote Sens.* **47**, 2284-2997 (2009).
15. Fornaro, G., Pauciullo, A., Reale, D. & Verde S. Multilook SAR Tomography for 3-D Reconstruction and Monitoring of Single Structures Applied to COSMO-SKYMED Data. *IEEE J. Sel. Topics Appl. Earth Observ. In Remote Sens.* **17**, 2776-2785 (2014).
16. Fornaro, G., Verde, S., Reale, D. & Pauciullo, A. An Approach Based on Covariance Matrix Decomposition to Improve Multibaseline-Multitemporal Interferometric SAR Processing. *IEEE Trans. Geosci. Remote Sens.* **54**, 2050-2065 (2015b).
17. Lohman, R. B. & Simons, M. Some thoughts on the use of InSAR data to constrain models of surface deformation: Noise structure and data downsampling. *Geochem. Geophys. Geosyst.* **6**, Q01007, 10.1029/2004GC000841 (2005).
18. Cheloni, D. *et al.* Coseismic and post-seismic slip of the 2009 L'Aquila (central Italy) Mw 6.3 earthquake and implications for seismic potential along the Campotosto fault from joint inversion of high-precision levelling, InSAR ad GPS data. *Tectonophysics* **622**, 168-185 (2014).
19. McCaffrey, R. Time-dependent inversion of three-component continuous GPS for steady and transient sources in northern Cascadia. *Geophys. Res. Lett.* **36,** L07304, 10.1029/2008GL036784 (2009).
20. Nabelek, J. Determination of earthquake source parameters from inversion of body waves. *Ph.D. Thesis* **262 pp.,** Mass. Inst. of Technol., Cambridge.
21. Okada, Y. Surface deformation due to shear and tensile faults in a half-space. *Bull. Seism. Soc. Am.* **75**, 1135-1154 (1985).
22. Stark, P. B. & Parker, R. L. Bounded-variable least-squares algorithm – An algorithm and implications. *Comput. Stat.* **10**, 129-141 (1995).
23. King, G. C. O, Stein, R. S., & Lin, J. Static stress changes and triggering of earthquakes. *Bull. Seism. Soc. Am.* **84,** 935-953 (1994).
24. Harris, R. A., & Simpson, R. W. In the shasod of 1857 – The effect of the Great Ft. Tejoin earthquake on subsequent earthquakes in southern California. *Geophys. Res. Lett.* **23,** 229-232 (1996).
25. Hamling, I. J. *et al.* Crustal deformation and stress transfer during a propagating earthquake sequence: The 2013 Cook Strait sequence, central New Zealand. *J. Geophys. Res.* **119,** 6080-6092, doi:10.1002/2014JB011084 (2014).
26. Lin, J. & Stein, R. S. Stress triggering in thrust and subduction earthquakes and stress interaction between the southern San Andreas and nearby thrust and strike-slip fault. *J. Geophys. Res.* **109,** B02303, doi:10.1029/2003JB002607 (2004).
27. Wessel, P. & Smith, W. H. F. New improved version of the generic mapping tools released. *Eos. Trans. AGU* **79**, 577-579 (1998).

2. Supplementary Tables

Supplementary Table T1. Coordinates and velocities of continuous GPS station in southern Italy. Coordinates, velocities (in Eurasia reference frame) and errors for each GPS station in Southern Italy processed and used for reference frame realization. Site name, latitude and longitude (in degrees), east and north component of the velocity and relative errors (in mm/yr), time of acquisition (start/end in decimal year), GPS network.

ACCA	15.3312	41.1586	1.20	3.80	0.17	0.20	2007.35-2016.33	PUGLIA
ACER	15.9424	40.7867	1.00	4.20	0.15	0.15	2007.53-2016.48	RING
ALBI	16.4559	39.9458	0.60	4.20	0.37	0.73	2012.36-2016.48	RING
ALTA	16.5586	40.8228	0.90	4.10	0.23	0.21	2011.42-2016.48	ITALPOS
AMUR	16.6040	40.9073	0.90	4.38	0.10	0.10	2005.62-2016.48	RING
ANG1	15.1839	40.9309	0.00	2.80	0.16	0.22	2008.51-2016.48	CAMPANIA
AQSA	16.0837	39.7210	0.40	3.90	0.32	0.37	2012.36-2016.00	RING
AV01	15.0827	41.1098	-0.50	2.73	0.19	0.21	2009.27-2014.75	ITALPOS
AV02	15.0217	40.8440	-0.50	2.27	0.34	0.37	2009.38-2014.93	ITALPOS
AV04	15.4386	40.9004	0.82	4.33	0.21	0.22	2009.60-2016.48	RING
BELV	15.8548	39.6181	0.30	3.10	0.38	0.38	2012.80-2016.48	NETGEO
BISI	16.2866	39.5135	0.86	3.60	0.17	0.17	2009.63-2016.40	CALABRIA
BULG	15.3777	40.0782	-0.20	2.40	0.11	0.14	2006.60-2016.48	RING
CADM	16.2737	41.0776	1.20	4.50	0.12	0.14	2000.51-2014.11	RING
CAFE	15.2366	41.0281	0.10	3.30	0.13	0.13	2005.87-2016.48	RING
CAMA	15.8506	40.6577	0.90	4.60	0.19	0.17	2007.51-2016.43	ISPRA
CASR	18.3473	39.8333	0.74	4.20	0.29	0.33	2012.05-2016.48	NETGEO
CASV	16.2049	39.8103	0.17	3.55	0.17	0.20	2010.08-2016.48	CALABRIA
CAVI	16.2132	39.8155	0.30	3.76	0.38	0.44	2012.10-2016.48	NETGEO
CDRU	15.3047	40.4897	-0.50	2.20	0.24	0.18	2005.67-2016.47	RING
CETR	15.9546	39.5287	0.40	3.17	0.17	0.21	2006.86-2016.09	UNAVCO
CMPR	15.3029	40.3179	-0.20	2.00	0.15	0.18	2005.68-2016.48	RING
COLR	16.4222	40.1934	0.80	4.40	0.21	0.19	2011.70-2016.48	RING
CRAC	16.4352	40.3814	0.80	4.30	0.12	0.14	2005.95-2016.48	RING
CUCC	15.8155	39.9938	-0.09	3.20	0.28	0.17	2005.68-2016.02	RING
DIMT	15.8218	39.6763	0.50	3.21	0.34	0.24	2010.41-2015.15	CALABRIA
FASA	17.3590	40.8348	1.10	4.20	0.14	0.13	2007.22-2016.47	PUGLIA
GINO	16.7578	40.5780	1.42	4.20	0.14	0.14	2007.22-2016.47	PUGLIA
GIUR	18.4300	40.1244	0.90	3.80	0.12	0.12	2007.22-2016.47	PUGLIA
GRO1	15.1009	41.0670	-0.20	2.70	0.13	0.15	2008.81-2016.48	RING
GROT	15.0599	41.0728	-0.10	2.60	0.10	0.12	2004.40-2016.48	RING
GRSN	16.2775	40.6333	0.70	3.70	0.28	0.42	2012.20-2016.48	ITALPOS
MAT1	16.7045	40.6491	1.00	4.00	0.09	0.14	2001.52-2016.48	IGS
MATA	16.5864	40.6785	0.55	4.66	0.26	0.35	2012.05-2016.48	NETGEO
MATE	16.7045	40.6491	0.80	4.40	0.09	0.12	2000.01-2016.48	IGS
MATG	16.7046	40.6490	1.10	4.60	0.22	0.39	2012.09-2016.48	ASI
MCEL	15.8015	40.3255	0.50	4.30	0.14	0.16	2006.60-2016.48	RING
MCRV	15.1681	40.7826	-1.30	1.80	0.37	0.27	2005.56-2016.48	RING
MLFT	16.6042	41.1956	0.70	4.00	0.18	0.21	2011.07-2016.37	ITALPOS
MMET	16.7864	40.3897	0.70	4.00	0.09	0.10	2004.40-2016.48	BASILICATA
MMNO	15.9721	39.8700	-0.80	1.80	0.34	0.23	2011.70-2016.48	RING
MNIA	16.6873	40.3649	0.40	3.70	0.21	0.27	2012.51-2016.47	ITALPOS
MOLF	16.5851	41.1844	0.74	4.47	0.27	0.32	2012.05-2016.36	NETGEO
MRLC	15.4887	40.7564	1.00	4.10	0.30	0.37	2004.90-2016.42	RING

MRVN 16.1959 41.0609 1.00 4.30 0.14 0.12 2006.38-2016.48 RING
MTSN 15.7512 40.2662 0.40 3.60 0.16 0.15 2006.60-2016.48 RING
NOCI 17.0644 40.7888 1.42 4.10 0.14 0.12 2005.57-2016.48 RING
PALZ 15.9602 40.9439 0.90 4.40 0.14 0.12 2006.61-2016.48 RING
PODE 15.7768 40.7805 0.80 4.10 0.32 0.37 2012.07-2016.48 NETGEO
POGG 16.2538 40.9166 0.80 4.10 0.15 0.16 2007.39-2016.47 PUGLIA
PRAI 15.7799 39.8968 0.17 2.80 0.40 0.20 2009.63-2016.08 CALABRIA
PTNZ 15.8170 40.6347 0.40 3.47 0.24 0.33 2011.41-2016.48 ITALPOS
PTRP 16.0608 40.5320 0.40 3.90 0.17 0.18 2007.50-2016.47 RING
PZSG 15.9868 40.9295 1.00 4.10 0.38 0.41 2012.05-2016.39 NETGEO
ROSC 16.6357 39.6004 1.40 3.90 0.39 0.51 2012.73-2016.48 NETGEO
ROSS 16.6405 39.5998 1.80 4.30 0.26 0.23 2009.63-2016.48 CALABRIA
SAL1 15.5566 40.4172 -0.50 2.79 0.47 0.29 2008.58-2016.48 CAMPANIA
SALB 16.3460 39.8770 1.10 4.20 0.22 0.20 2009.49-2016.38 RING
SAPR 15.6301 40.0736 -0.20 3.08 0.21 0.22 2008.23-2016.48 CAMPANIA
SASA 17.9646 40.3852 1.00 3.90 0.10 0.12 2007.22-2016.47 PUGLIA
SCHR 16.0853 40.1902 0.20 3.98 0.17 0.16 2005.71-2015.60 RING
SCTE 18.4672 40.0723 0.80 3.70 0.09 0.11 2006.09-2016.48 RING
SENS 16.3124 40.1388 1.50 4.00 0.27 0.37 2012.05-2016.48 NETGEO
SGTA 15.3653 41.1356 0.90 3.89 0.17 0.18 2005.98-2016.37 RING
SIRI 15.8664 40.1835 1.40 4.20 0.16 0.16 2006.60-2016.48 RING
SLCN 15.6330 40.3911 -0.40 2.20 0.28 0.21 2005.42-2016.48 RING
SNAL 15.2092 40.9255 0.10 2.80 0.12 0.15 2004.63-2016.48 RING
SVTO 16.4405 40.6044 0.90 4.10 0.12 0.16 2006.64-2016.48 RING
TARA 17.2835 40.5266 0.80 4.40 0.15 0.20 2010.97-2016.48 ITALPOS
TITO 15.7237 40.6013 0.70 4.20 0.17 0.17 2002.00-2016.48 RING
TRE2 16.5269 39.8691 1.20 4.20 0.19 0.20 2009.63-2016.29 CALABRIA
UGEN 18.1620 39.9277 0.80 4.00 0.12 0.14 2007.37-2016.47 PUGLIA
USAL 18.1115 40.3349 1.10 4.10 0.14 0.15 2010.15-2016.48 EUREF
VAL1 16.9045 41.0164 1.30 4.30 0.14 0.15 2007.54-2016.47 PUGLIA
VENO 15.8087 40.9667 0.96 4.15 0.19 0.30 2010.29-2016.48 ITALPOS
VIGG 16.1161 39.9861 1.70 4.60 0.81 0.60 2011.71-2015.67 RING
VULT 15.6164 40.9549 0.90 4.12 0.12 0.15 2005.63-2016.48 RING

Supplementary Table T2. Relative block angular velocities. Latitude and longitude (in degrees). Ω is the rotation rate in degrees/Ma (the first block rotates counter-clockwise relative to the second); $\mathbf{S}_{\Omega}$ is the formal error (1-sigma confidence level).

Block pair	Lat.	Long.	Ω	$\mathbf{S}_{\Omega}$
Ap-Eu	36.418	33.552	-0.163	0.020

Supplementary Table T3. CSK acquisition parameters in terms of temporal and spatial baselines. Record number, satellite, acquisition time (year/month/day), temporal baseline (in days), spatial baseline (in m). In bold the master image (T22).

#	Record Number	Sat ID	Acquisition Date	Temporal Baseline (days)	Spatial Baseline (m)
T00	100803247	CSKS4	2012/06/05	-352	449.370
T01	100820263	CSKS4	2012/07/23	-304	524.418
T02	100826385	CSKS4	2012/08/08	-288	250.959
T03	100832190	CSKS4	2012/08/24	-272	217.629
T04	100849423	CSKS4	2012/10/11	-224	296.055
T05	100863640	CSKS4	2012/11/12	-192	-496.762
T06	100871068	CSKS1	2012/12/02	-172	975.786
T07	100876223	CSKS1	2012/12/18	-156	437.414
T08	100878887	CSKS2	2012/12/26	-148	512.200
T09	100881523	CSKS1	2013/01/03	-140	-189.659
T10	100884717	CSKS2	2013/01/11	-132	146.171
T11	100887321	CSKS1	2013/01/19	-124	511.459
T12	100890398	CSKS2	2013/01/27	-116	-196.854
T13	100893762	CSKS1	2013/02/04	-108	651.344
T14	100897396	CSKS2	2013/02/12	-100	-201.600
T15	100900278	CSKS1	2013/02/20	-92	-505.324
T16	100903849	CSKS2	2013/02/28	-84	-374.943
T17	100906577	CSKS1	2013/03/08	-76	249.971
T18	100914104	CSKS1	2013/03/24	-60	278.830
T19	100917848	CSKS2	2013/04/01	-52	-240.065
T20	100926831	CSKS4	2013/04/21	-32	370.487
T21	100933226	CSKS4	2013/05/07	-16	-328.413
**T22**	**100939378**	**CSKS4**	**2013/05/23**	**0**	**0.00000**
T23	100945665	CSKS4	2013/06/08	16	-316.845
T24	100952700	CSKS4	2013/06/24	32	480.136
T25	100959341	CSKS4	2013/07/10	48	-145.216
T26	100965170	CSKS4	2013/07/26	64	-407.433
T27	100971809	CSKS4	2013/08/11	80	212.124
T28	100979018	CSKS4	2013/08/27	96	-366.143
T29	101002511	CSKS4	2013/10/14	144	-392.263
T30	101008385	CSKS4	2013/10/30	160	-240.787
T31	101015207	CSKS4	2013/11/15	176	794.984
T32	101022205	CSKS4	2013/12/01	192	-129.480
T33	101035864	CSKS4	2014/01/02	224	-354.448
T34	101042445	CSKS4	2014/01/18	240	-293.915
T35	101049126	CSKS4	2014/02/03	256	-718.847
T36	101055292	CSKS4	2014/02/19	272	-550.822
T37	101067171	CSKS4	2014/03/23	304	-919.733
T38	101074126	CSKS4	2014/04/08	320	-462.669

Supplementary Table T4. Transient GPS displacements. Latitude and longitude (in degrees). D_E, D_N are the east and north components of displacement in mm; S_E, S_E are the formal error (1-sigma confidence level).

GPS site	Lat.	Long.	D_E	D_N	S_E	S_E
AQSA	16.0839	39.7210	-0.07	0.08	1.00	1.00
CASV	16.2050	39.8100	0.63	-0.02	1.00	1.00
CAVI	16.2130	39.8150	0.70	-0.02	1.00	1.00
COLR	16.4220	40.1930	0.25	0.16	1.00	1.00
CUCC	15.8169	39.9940	-0.46	-0.11	1.00	1.00
DIMT	15.8220	39.6760	-0.48	-0.47	1.00	1.00
MMNO	15.9720	39.8700	-6.33	-4.23	1.00	1.00
PRAI	15.7799	39.8970	-1.88	-0.29	1.00	1.00
SALB	16.3460	39.8770	0.71	0.07	1.00	1.00
SCHR	16.0850	40.1900	0.14	0.05	1.00	1.00
SENS	16.3120	40.1390	0.38	0.26	1.00	1.00
VIGG	16.1160	39.9860	2.01	1.33	1.00	1.00

Supplementary Table T5. GPS velocities in Apulia reference frame. Latitude and longitude (in degrees). V_E, V_N are the east and north components of velocity in mm/yr; S_E, S_E are the formal error (1-sigma confidence level).

GPS site	Lat.	Long.	V_E	V_N	S_E	S_E
AQSA	16.0839	39.7210	-0.06	-0.62	0.54	0.54
CASV	16.2050	39.8100	-0.47	-0.75	0.42	0.42
CAVI	16.2130	39.8150	-0.63	-0.70	0.52	0.52
COLR	16.4220	40.1930	-0.08	0.13	0.49	0.49
CUCC	15.8169	39.9940	-1.23	-1.16	0.32	0.32
DIMT	15.8220	39.6760	-0.30	-1.19	0.46	0.46
MMNO	15.9720	39.8700	-1.61	-1.65	0.49	0.49
PRAI	15.7799	39.8970	-0.81	-1.57	0.40	0.40
SALB	16.3460	39.8770	0.30	0.01	0.40	0.40
SCHR	16.0850	40.1900	-0.33	-0.50	0.32	0.32
SENS	16.3120	40.1390	0.60	-0.06	0.51	0.51
VIGG	16.1160	39.9860	1.02	0.60	0.50	0.50

3. Supplementary Figures

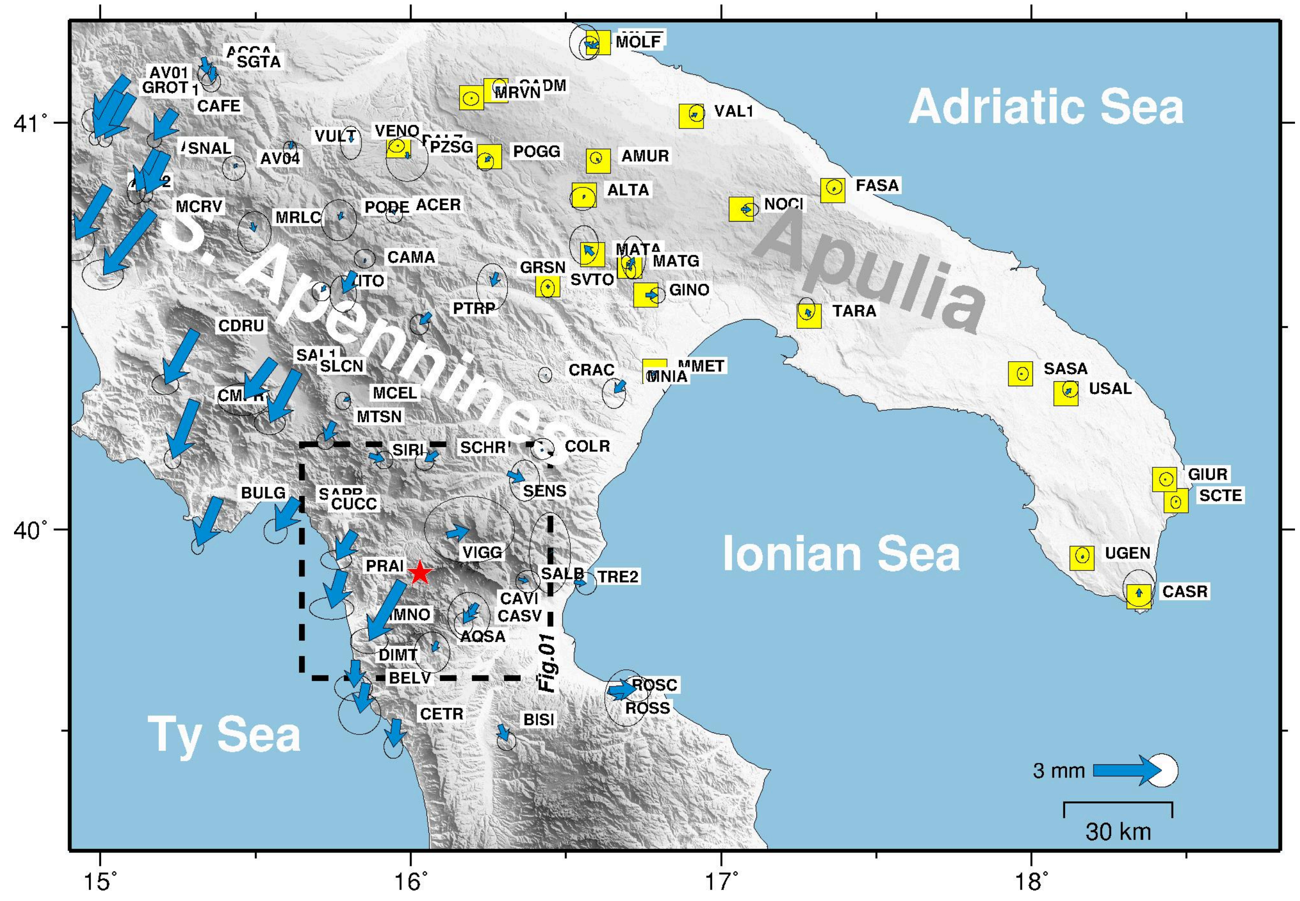

Supplementary Figure S1. GPS velocity field for the Pollino range and surrounding regions in an Apulia frame. Blue arrows are the horizontal velocity with error ellipses at 95% confidence interval. The red star is the 12 October 2012 M_W 5.1 main shock of the 2010-2014 Pollino swarm sequence. Site used to define the rotation of the Apulia (Ap) microplate are shown with yellow squares. The dashed lines enclose the study area. The map was created by using Genetic Mapping Tools software (GMT v4.5.14; http://gmt.soest.hawaii.edu/)[27].

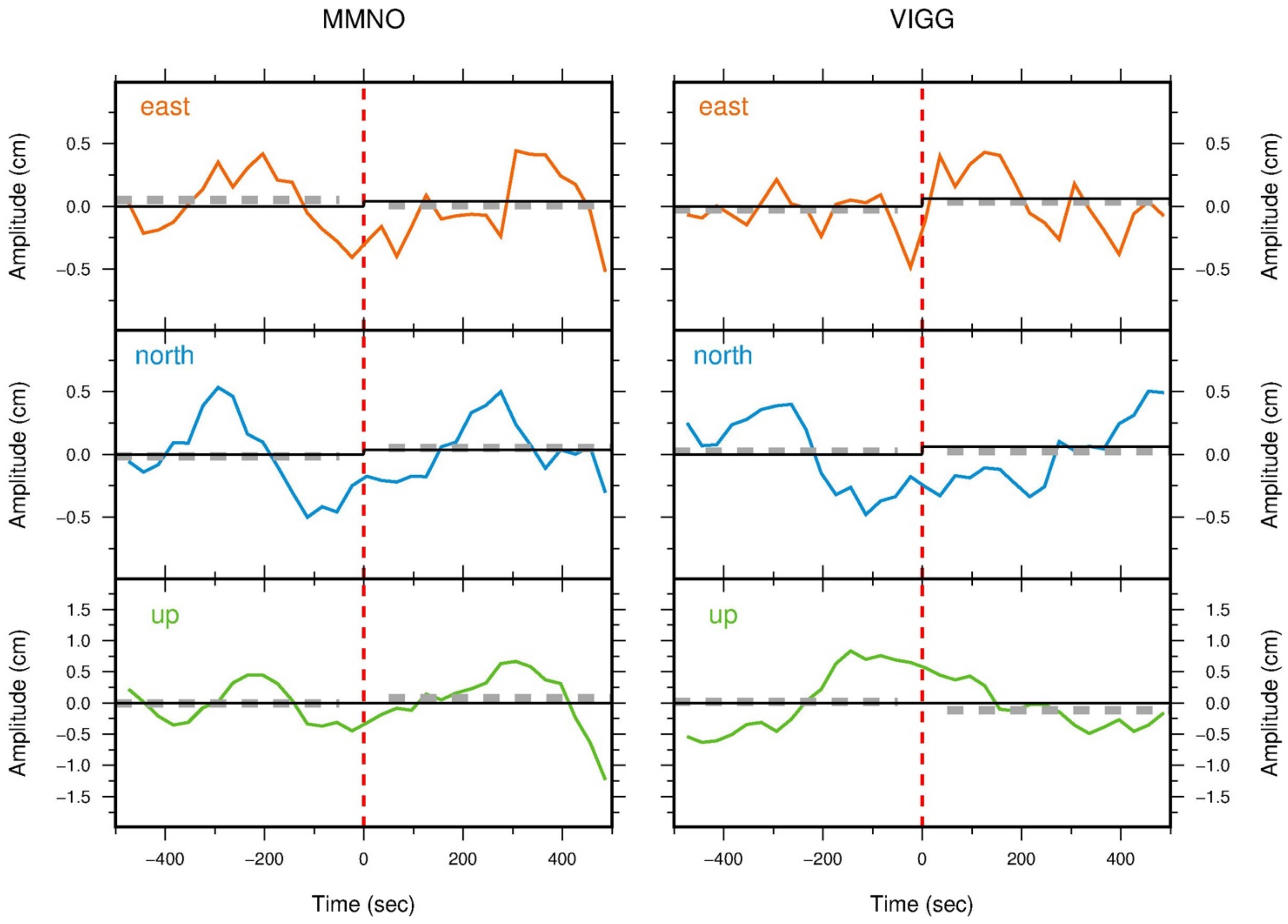

Supplementary Figure S2. High-rate GPS analysis. Horizontal and vertical components of 5 Hz GPS record at stations MMNO and VIGG. The orange, blue and green trends represent the east, north and vertical components, respectively. The grey dashed lines represent the mean position pre- and after the event, while thin solid black lines are the predicted offsets by our modelling of the M_W 5.1 event. Note that the predicted offsets are almost equal to zero. The red dashed line represents the time of the M_W 5.1 earthquake. The figures were created by using Genetic Mapping Tools software (GMT v4.5.14; http://gmt.soest.hawaii.edu/)[27].

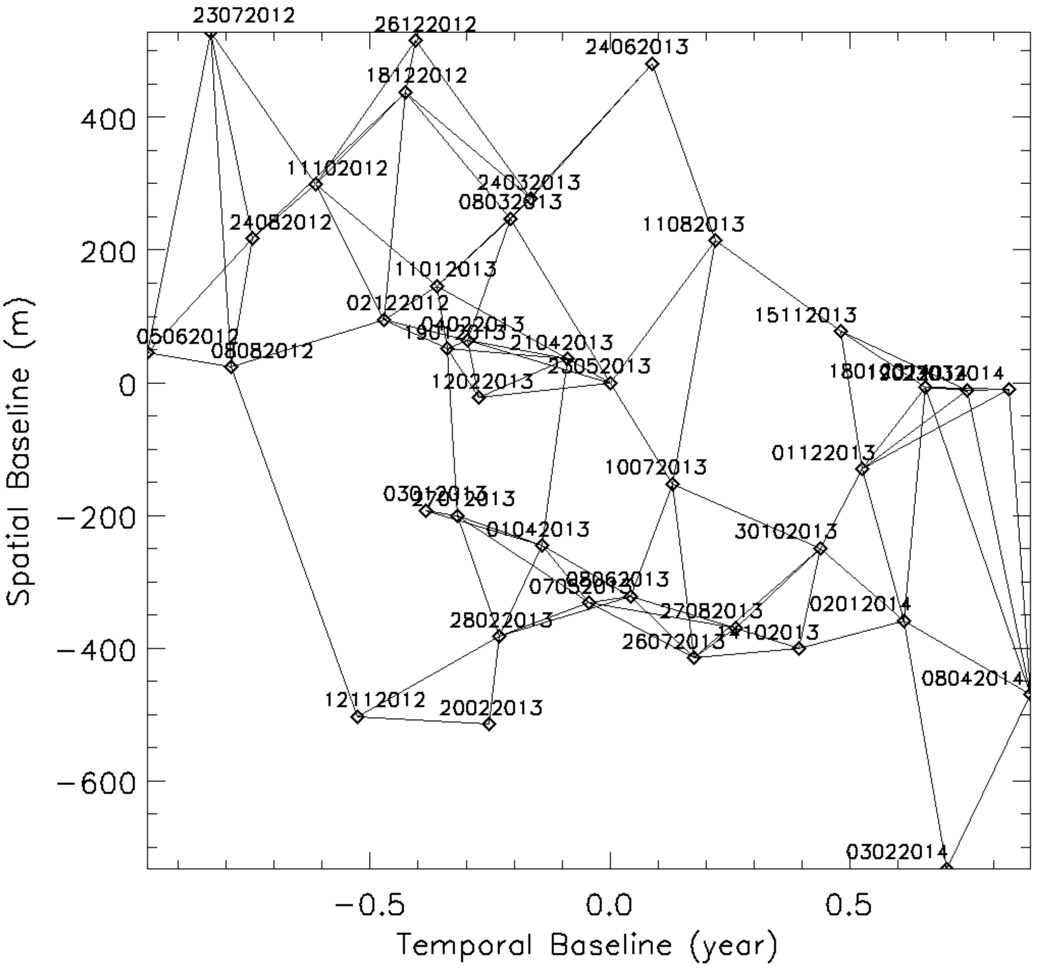

Supplementary Figure S3. Spatial and temporal baseline distribution of the CSK data set. The data set starts on 6 June 2012 and includes about 2 years of deformation. The lines between the different acquisitions define the interferometric pairs used for the construction of the SBAS time series. Master image is at coordinates (0,0).

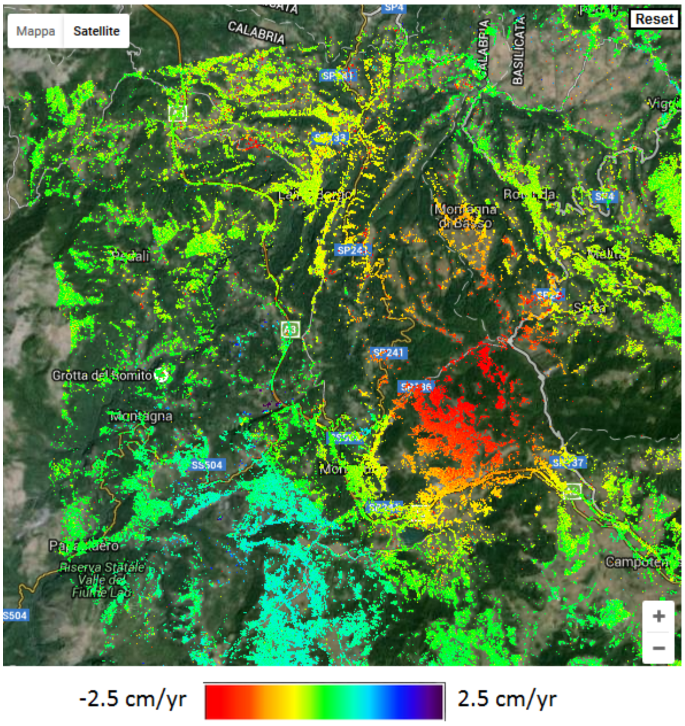

Supplementary Figure S4. Overlay of the deformation mean velocity of monitored scatterers to a Google Map image. Images ©2016 Google, Map data ©2016 Google.

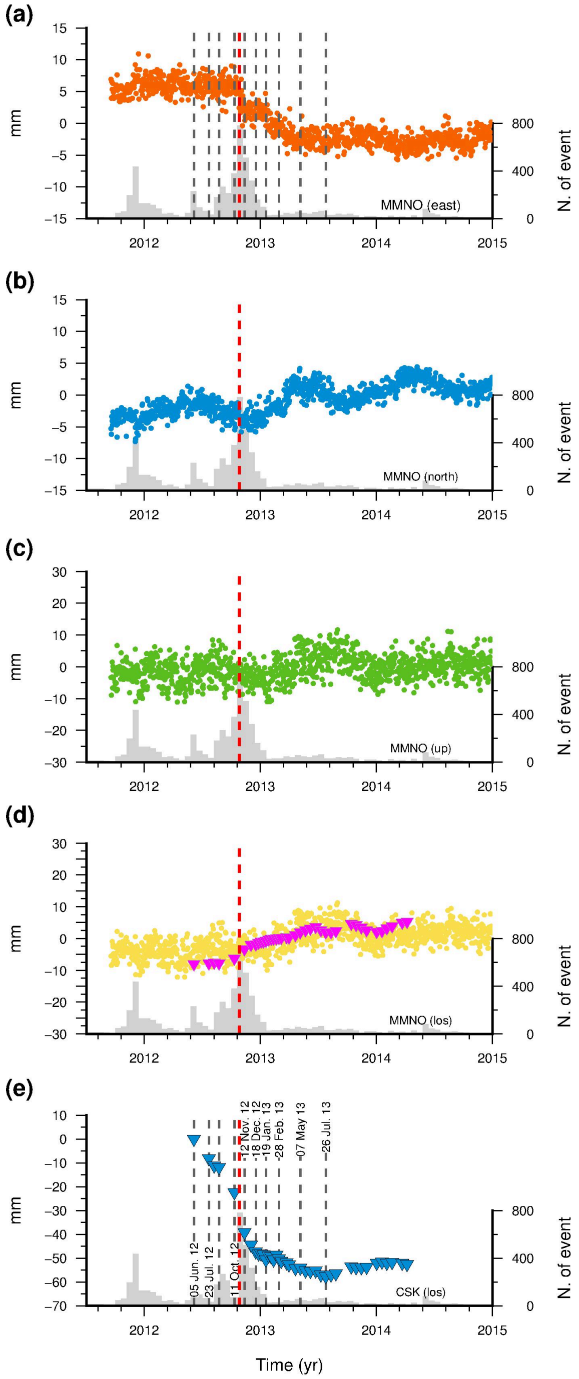

Supplementary Figure S5. Geodetic time series and seismicity rate. (**a**) Orange, (**b**) blue and (**c**) green dots indicate the east, north and vertical displacements, respectively, recorded at GPS site MMNO expressed with respect to the Apulian (Ap) reference frame. (**d**) Comparison of line-of-sight projected GPS time series (yellow circles) and DInSAR CSK time series (purple triangles). (**e**) CSK time series of selected points (blue triangles) in the area of maximum deformation (i.e., between MMNO and VIGG sites). We also plot the number of earthquakes (grey bars) as a function of time. The red dashed line indicates the 25 October 2012 M_W 5.1 main event of the swarm sequence, while the grey dashed lines show the main accelerations observed in the CSK time series (with labelled dates). The figures were created by using Genetic Mapping Tools software (GMT v4.5.14; http://gmt.soest.hawaii.edu/)[27].

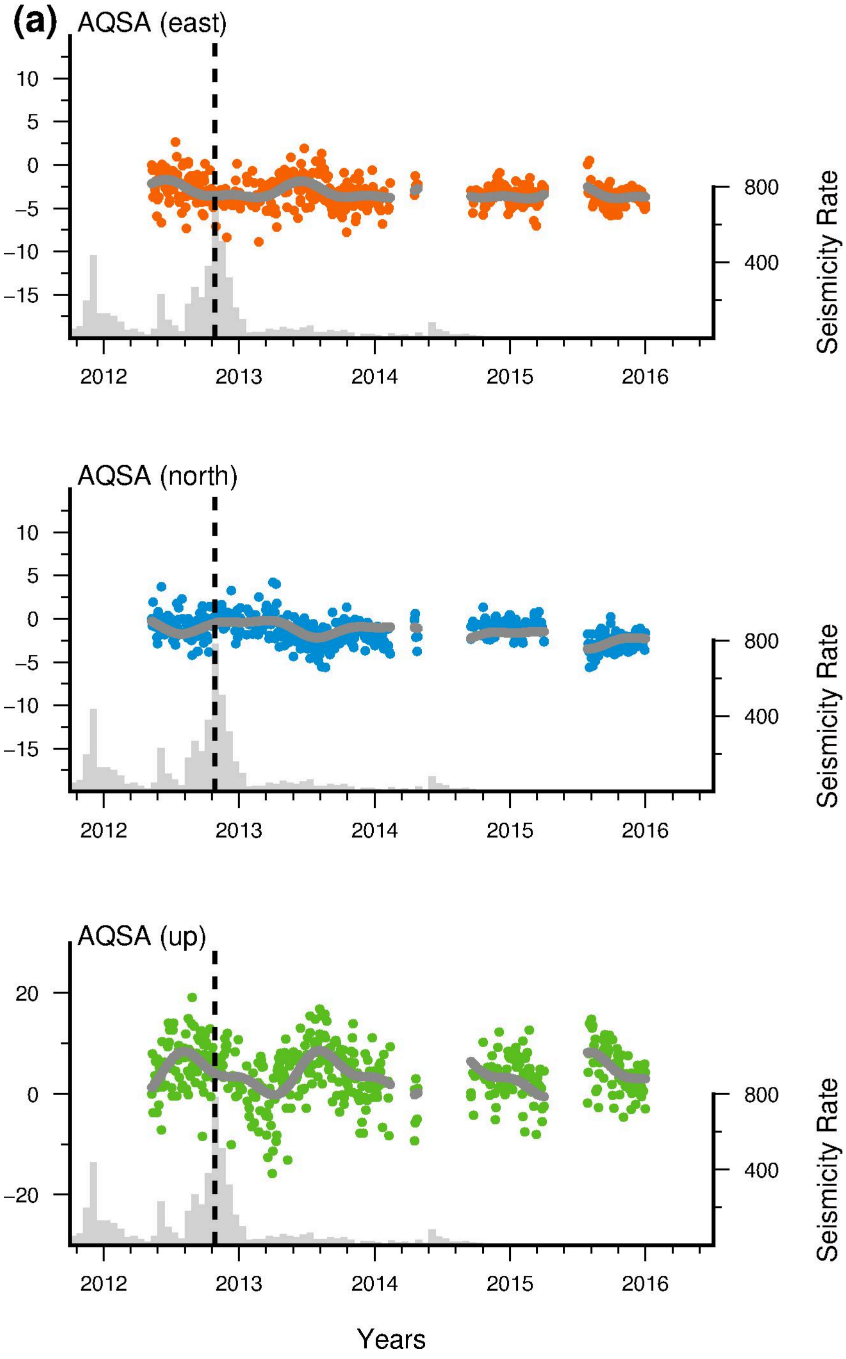

Supplementary Figure S6. Complete set of GPS time series. (**a**) AQSA station. (**b**) CASV. (**c**) CAVI. (**d**) COLR. (**e**) CUCC. (**f**) DIMT. (**g**) MMNO. (**h**) PRAI. (**i**) SALB. (**j**) SCHR. (**k**) SENS. (**l**) VIGG. GPS time series of all the sites used in the present study in an Apulia (Ap) reference frame, plotted with the distribution of seismicity. The circles (orange = E-component; blue = N-component; green = Up-component) are the daily positions, the solid grey curves the predicted positions by our preferred model, and the black dashed line the time of the 25 October 2012 M_W 5.1 earthquake. The grey histograms show the seismicity rate. The figures were created by using Genetic Mapping Tools software (GMT v4.5.14; http://gmt.soest.hawaii.edu/)[27].

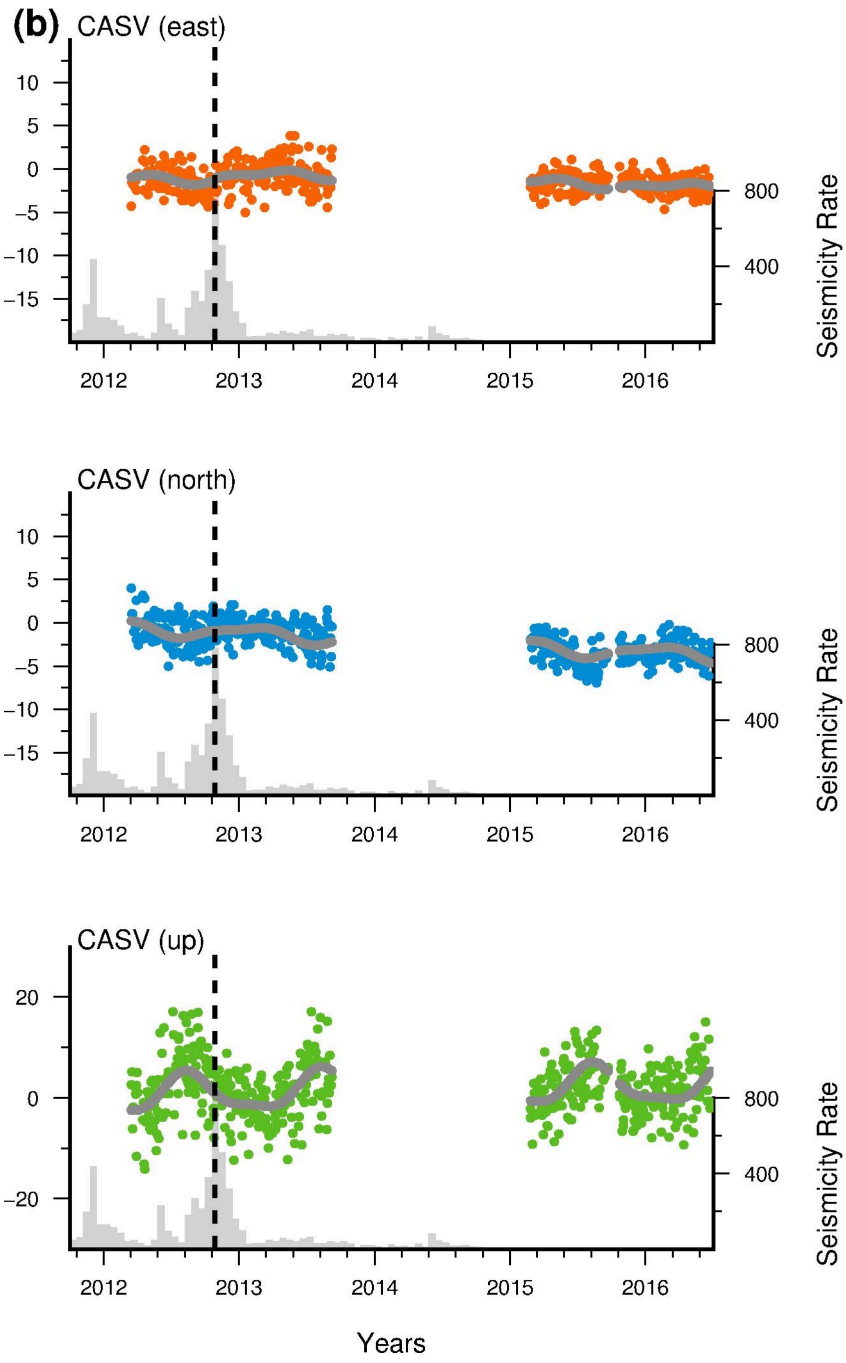

Supplementary Figure S6. (continued)

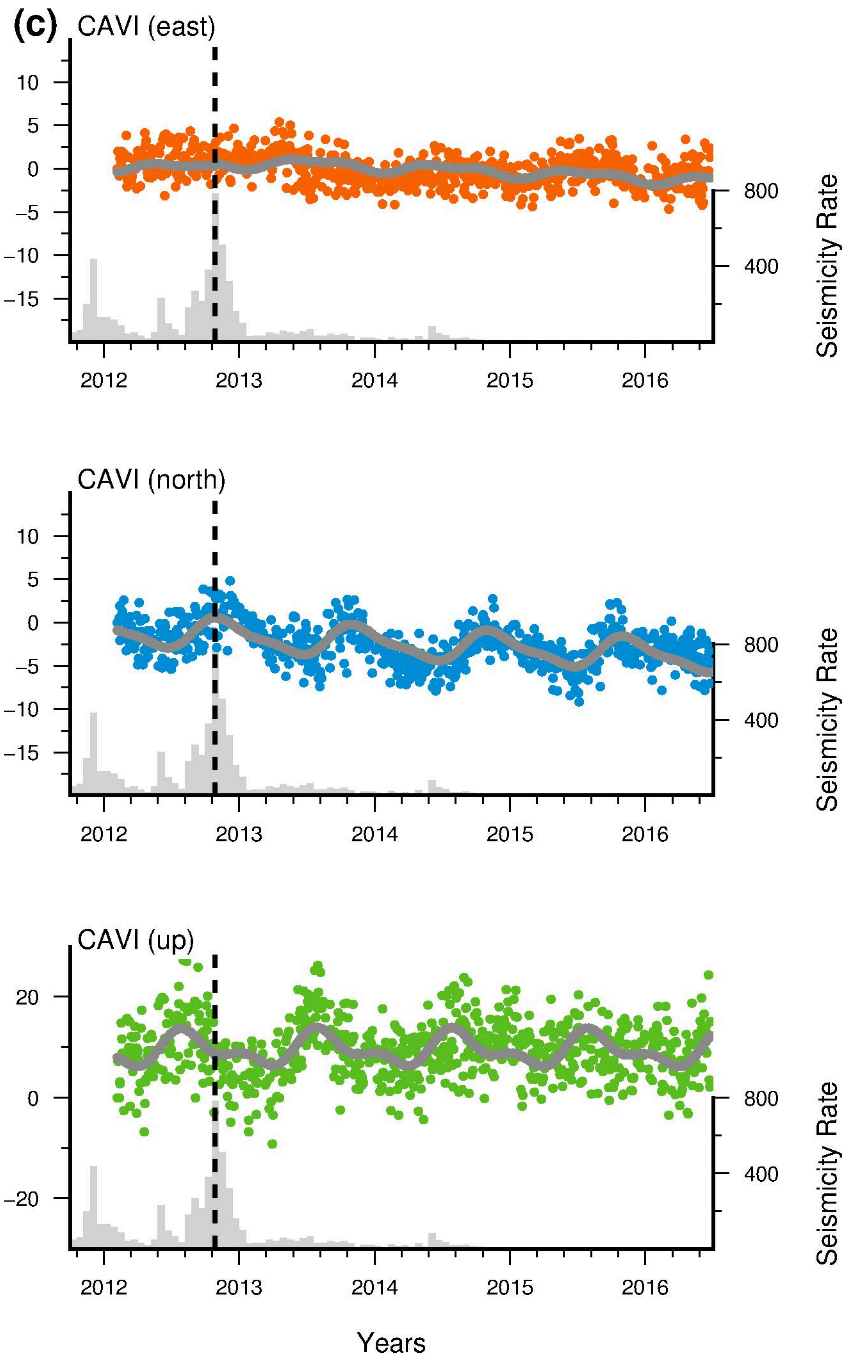

Supplementary Figure S6. (continued)

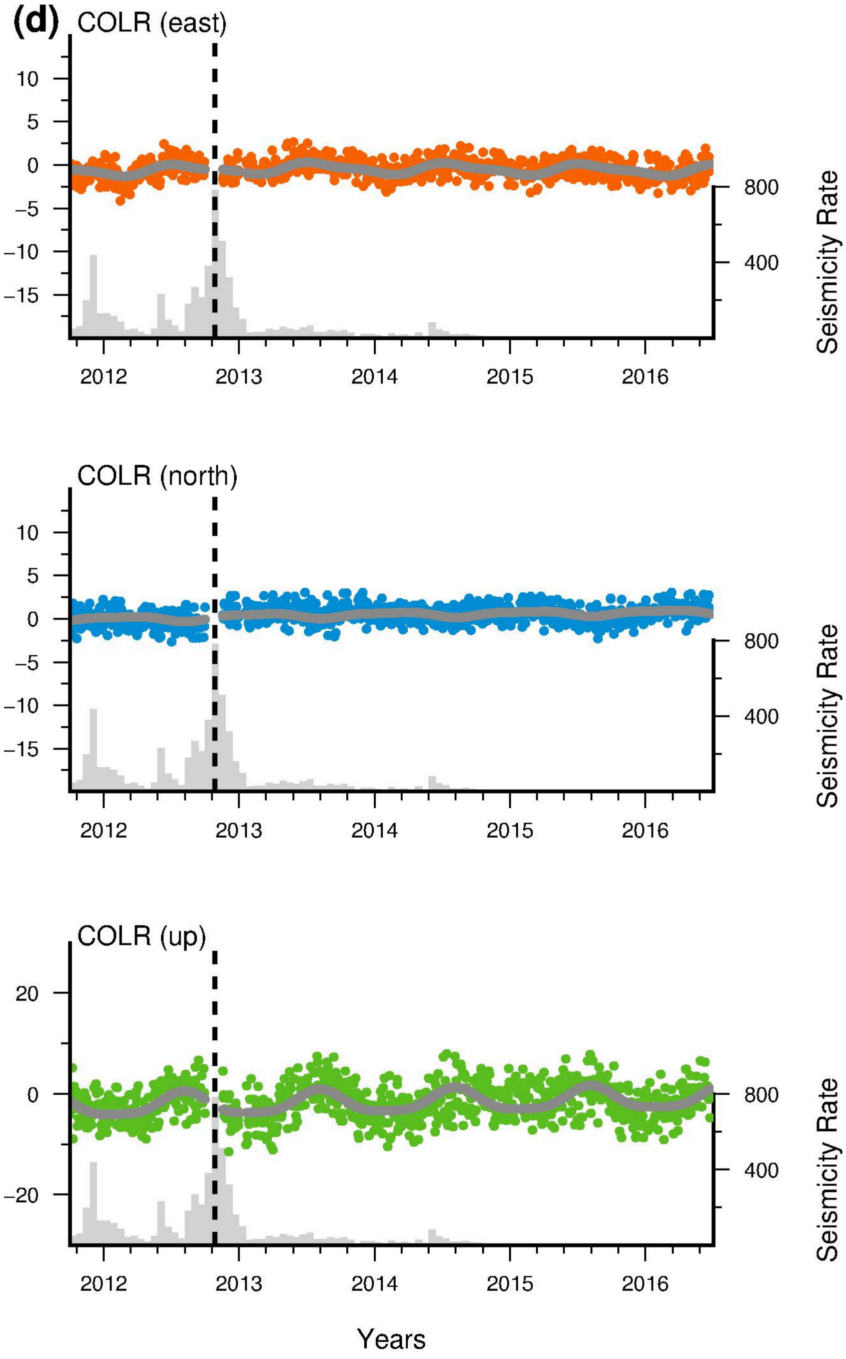

Supplementary Figure S6. (continued)

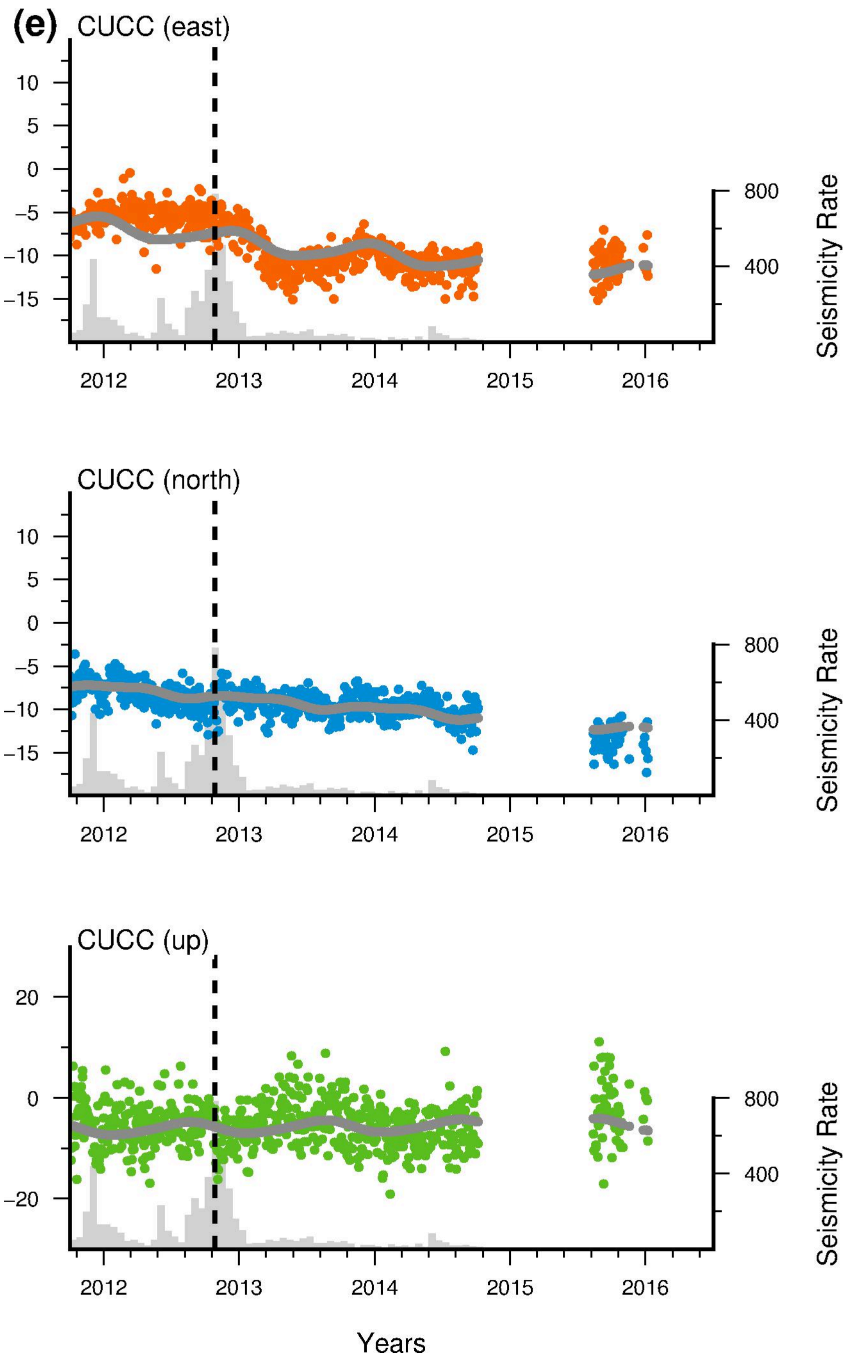

Supplementary Figure S6. (continued)

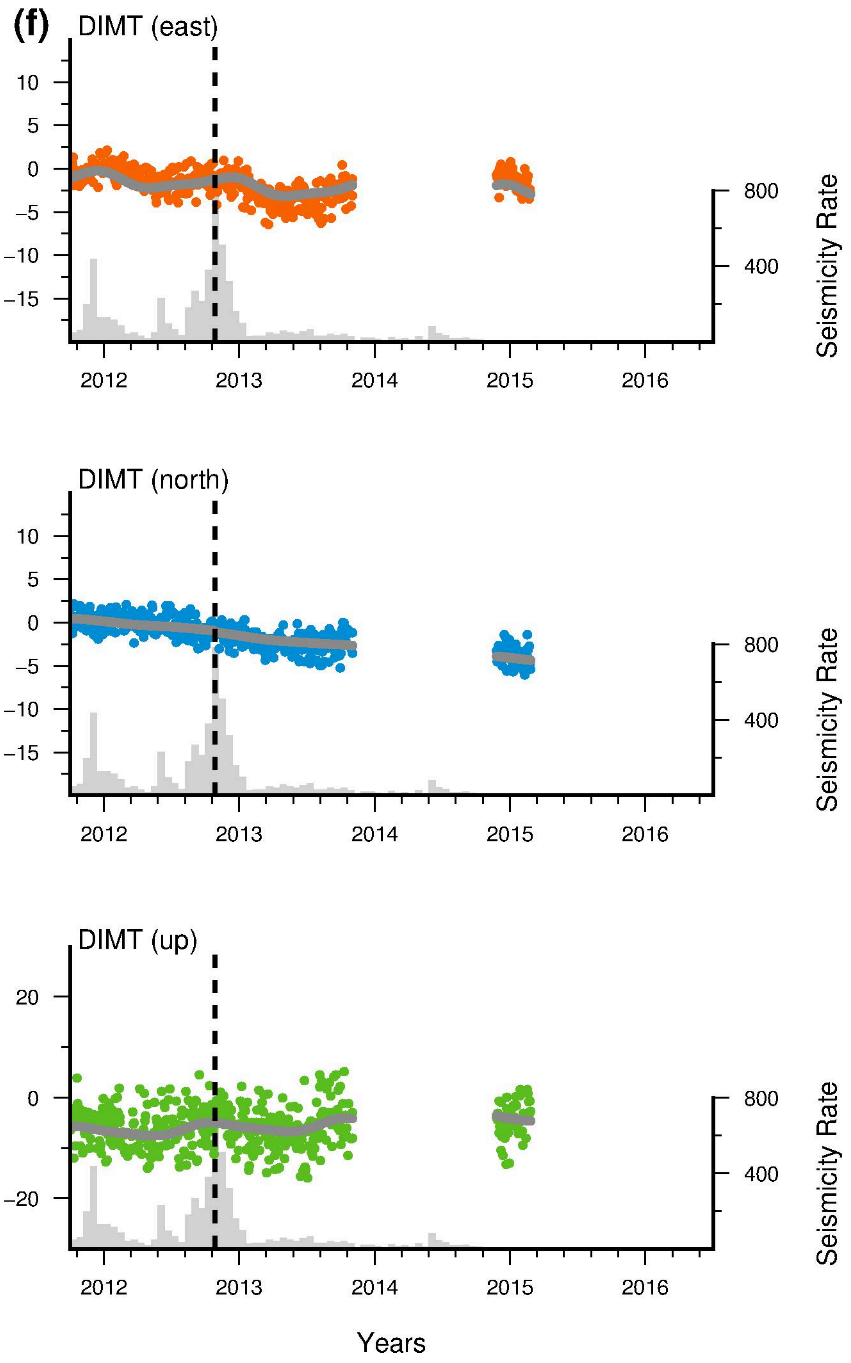

Supplementary Figure S6. (continued)

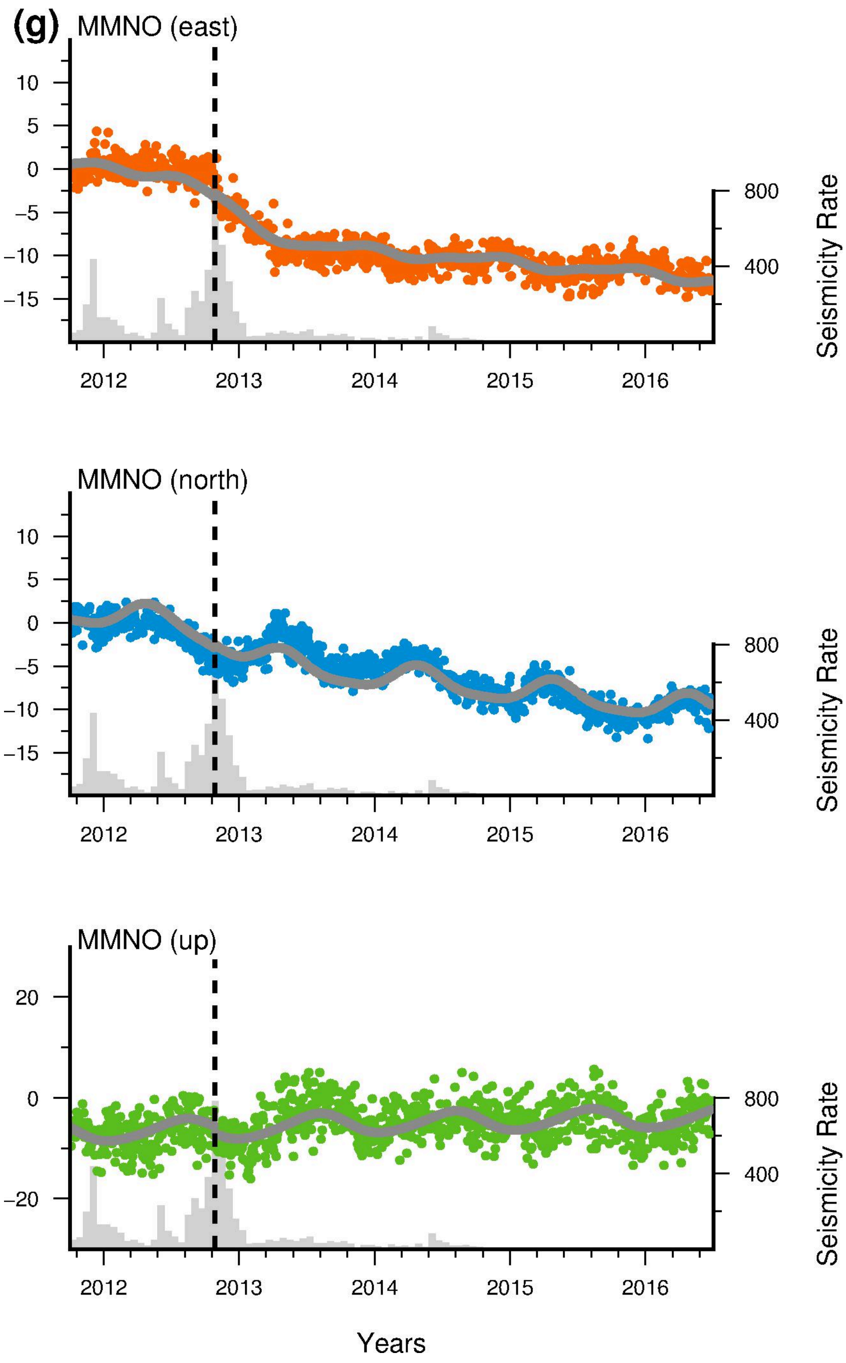

Supplementary Figure S6. (continued)

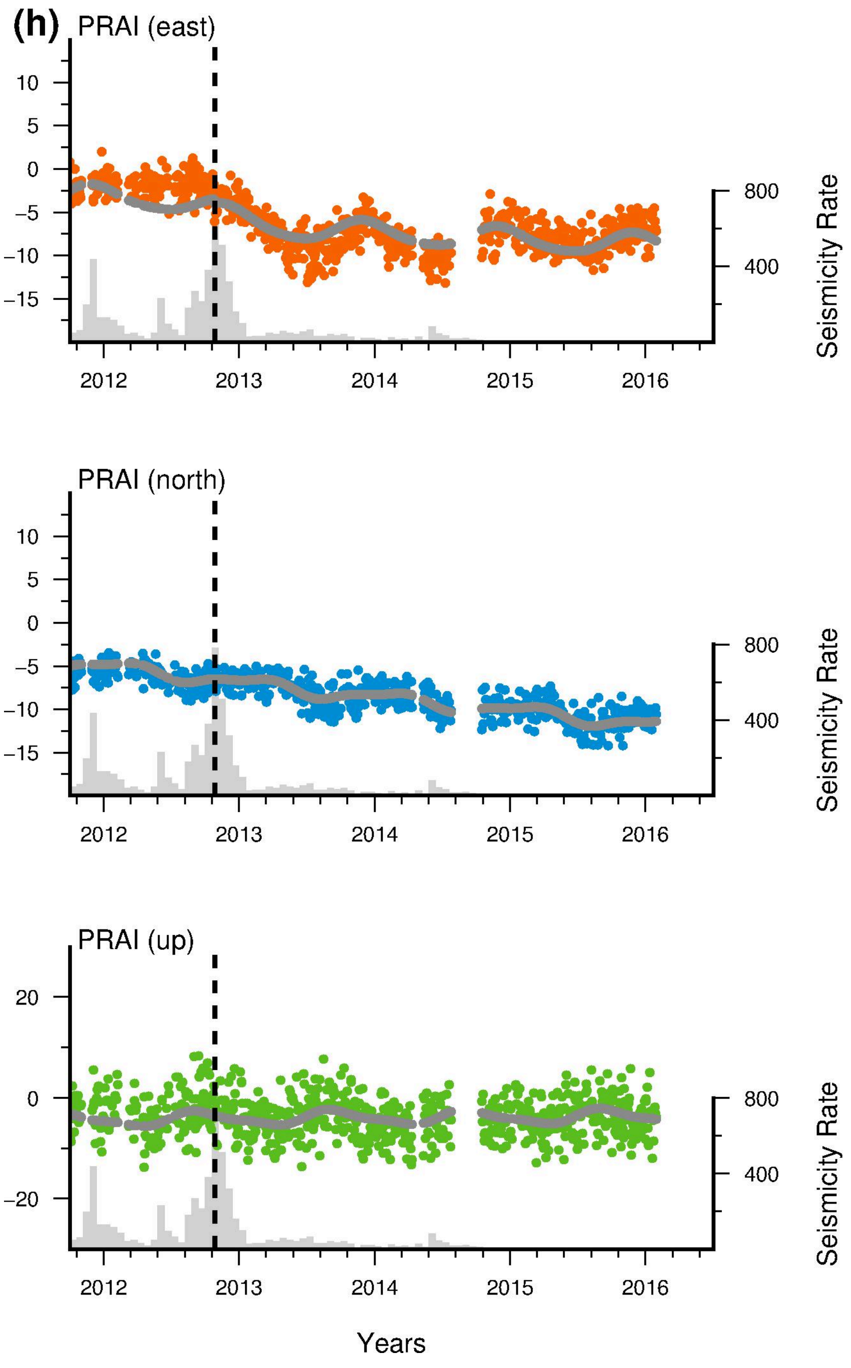

Supplementary Figure S6. (continued)

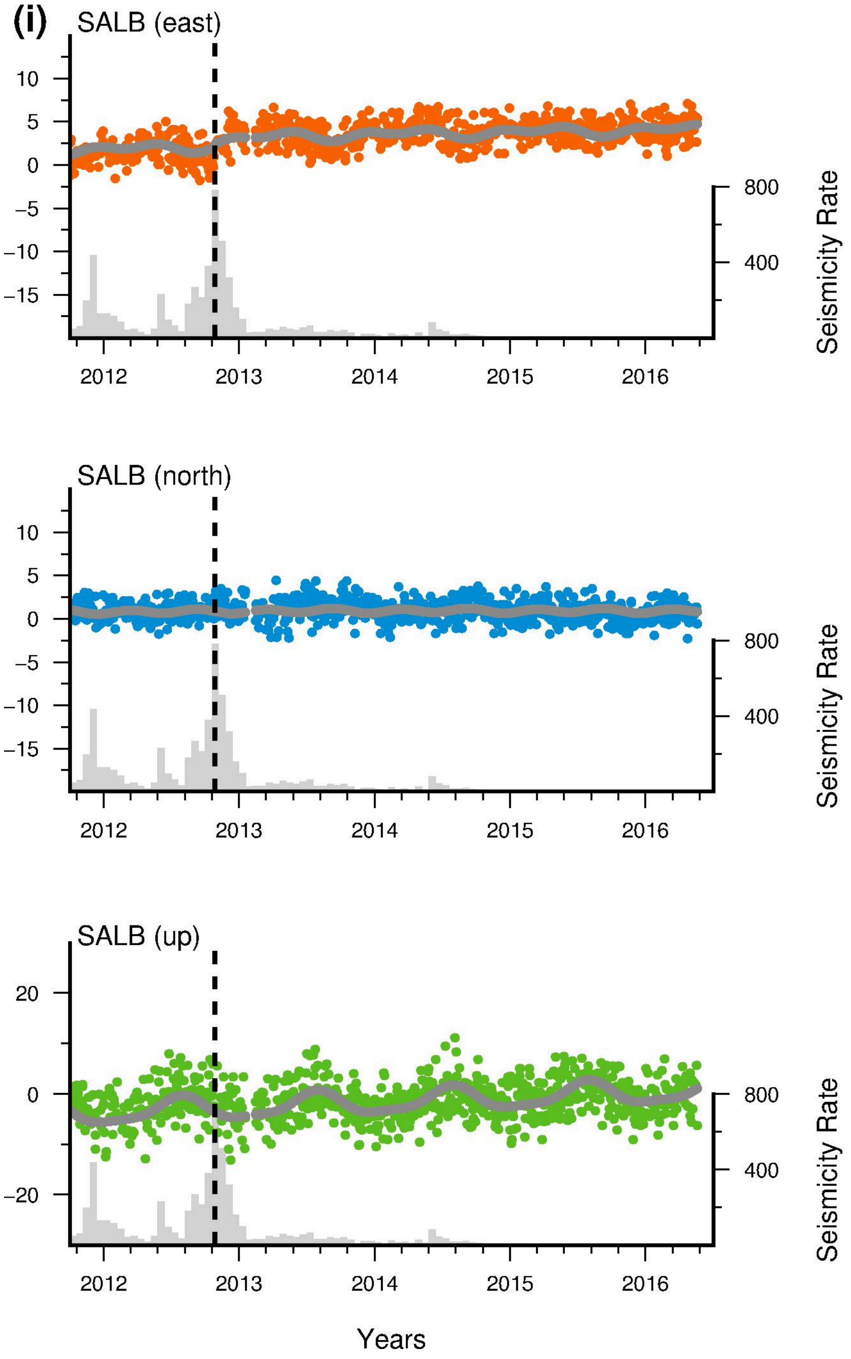

Supplementary Figure S6. (continued)

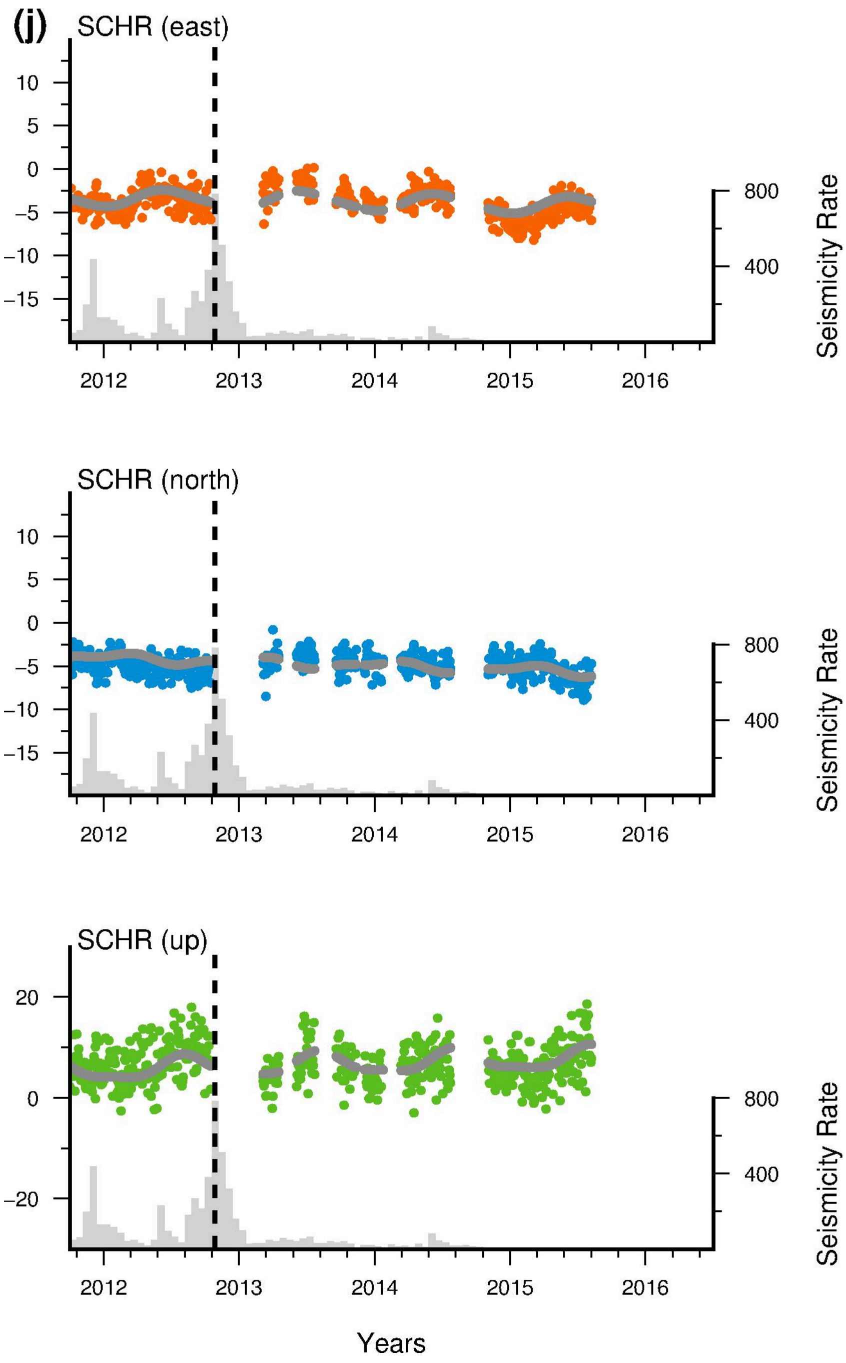

Supplementary Figure S6. (continued)

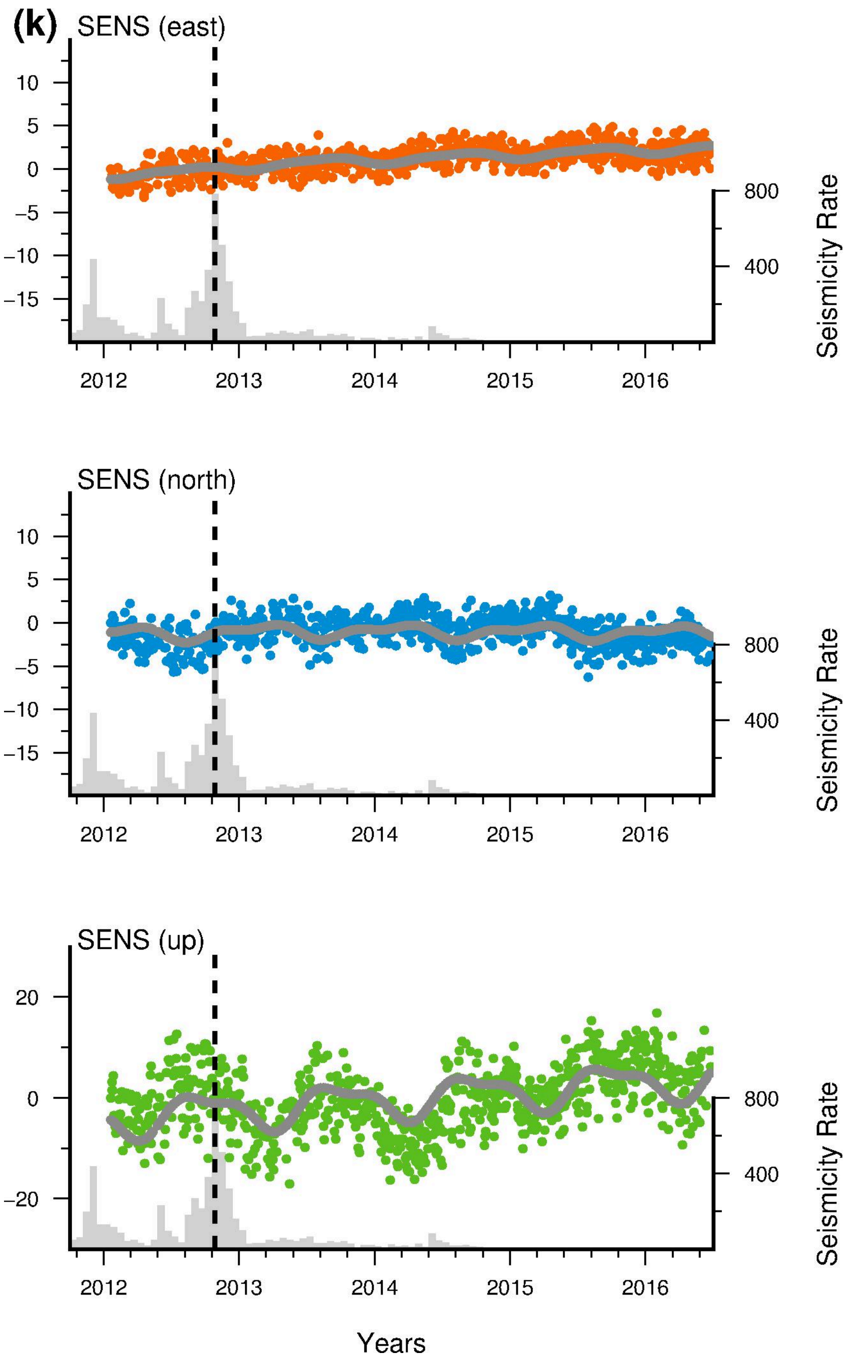

Supplementary Figure S6. (continued)

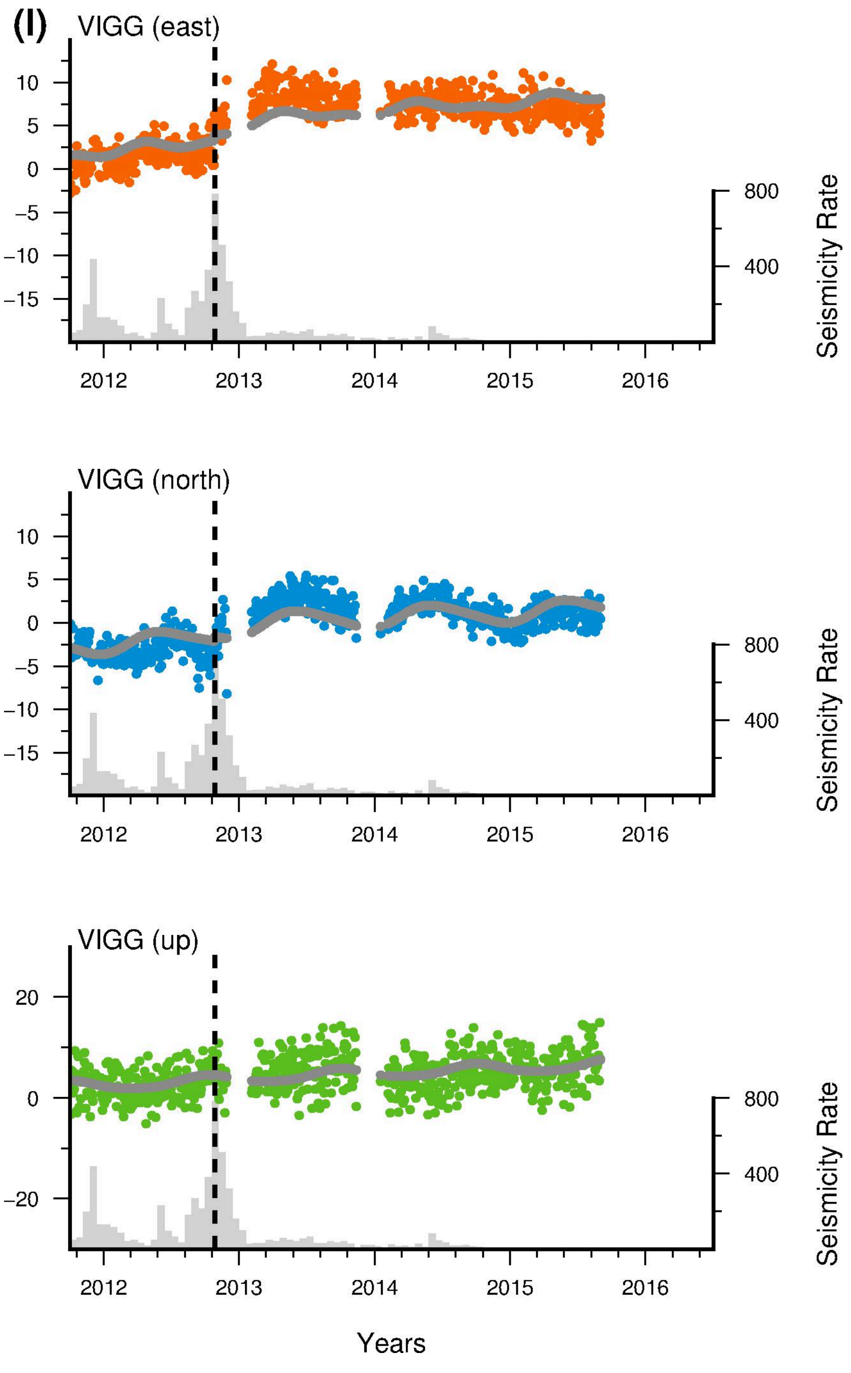

Supplementary Figure S6. (continued)

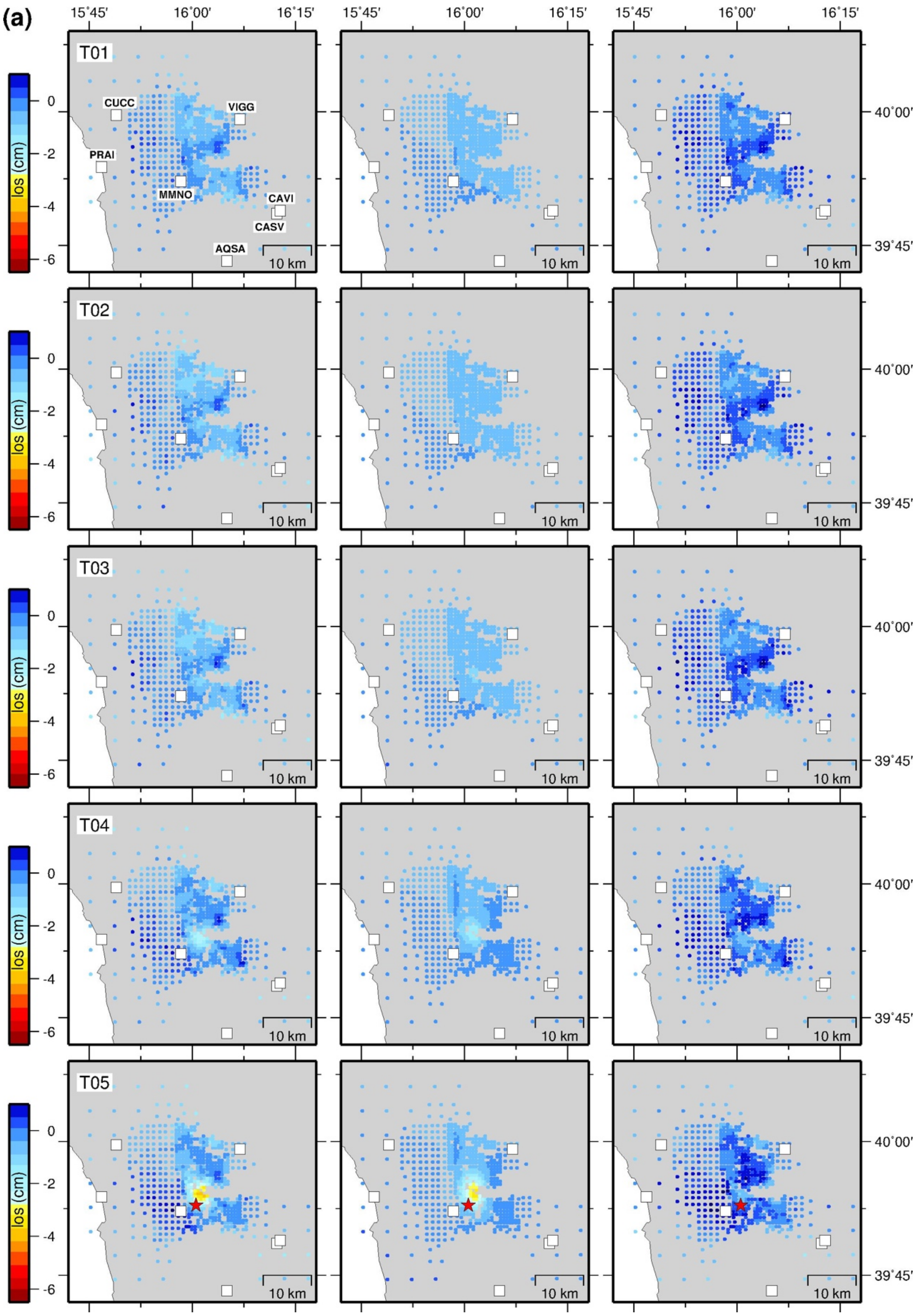

Supplementary Figure S7. Inverted InSAR interferograms. (**a**) T01-T05. (**b**) T06-T10. (**c**) T11-T15. (**d**) T16-T20. (**e**) T20-T25. (**f**) T25-T30. (**g**) T31-T35. Data (left panels), model (central panes), and residual (right panels) sampled points from all the unwrapped interferograms encompassing the Pollino swarm sequence from the CSK ascending track. The star indicates the location of the M_W 5.1 earthquake. Other symbols as in Figure 1. See Supplementary Table T3 for acquisition times. The maps were created by using Genetic Mapping Tools software (GMT v4.5.14; http://gmt.soest.hawaii.edu/)[27].

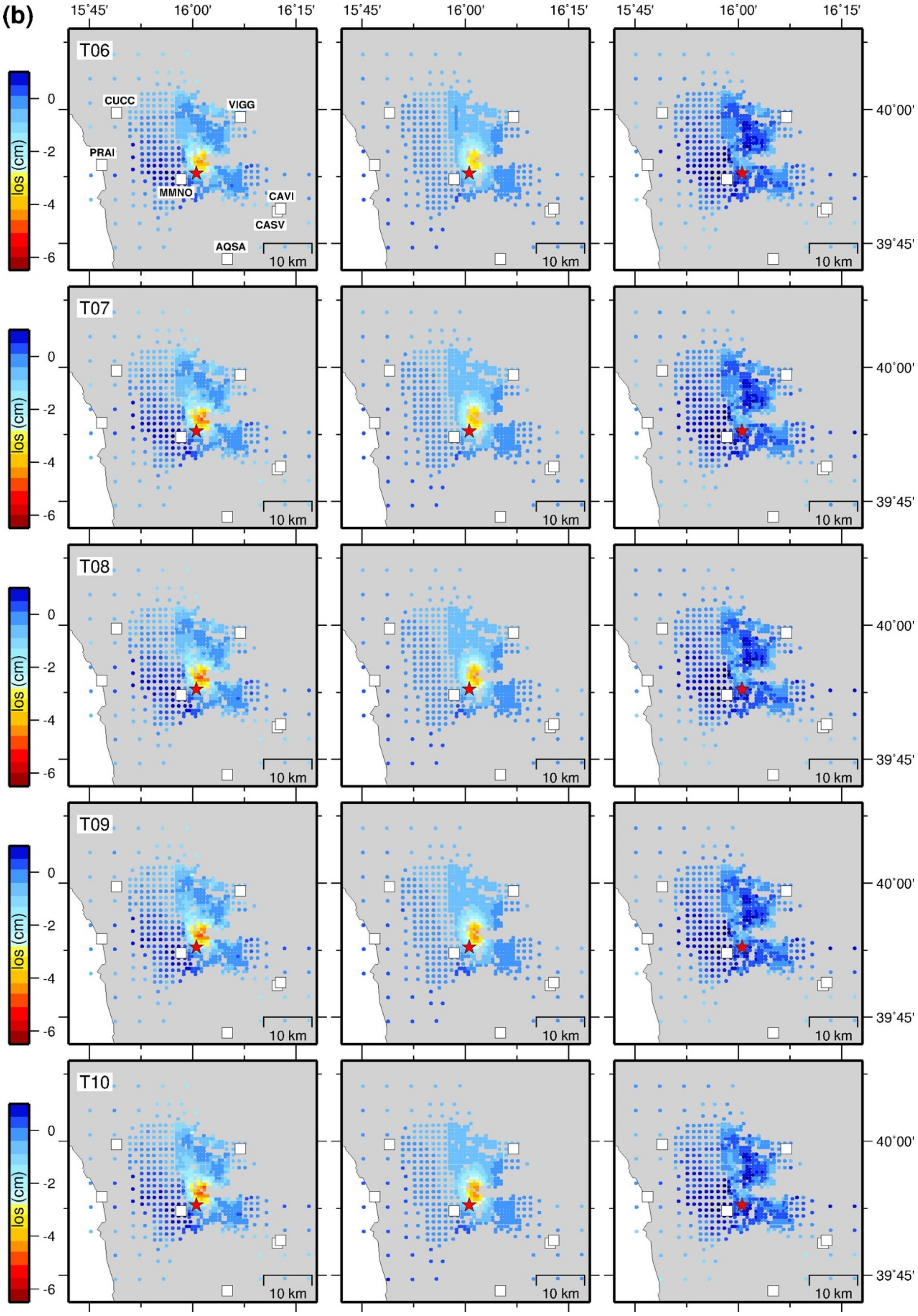

Supplementary Figure S7. (continued)

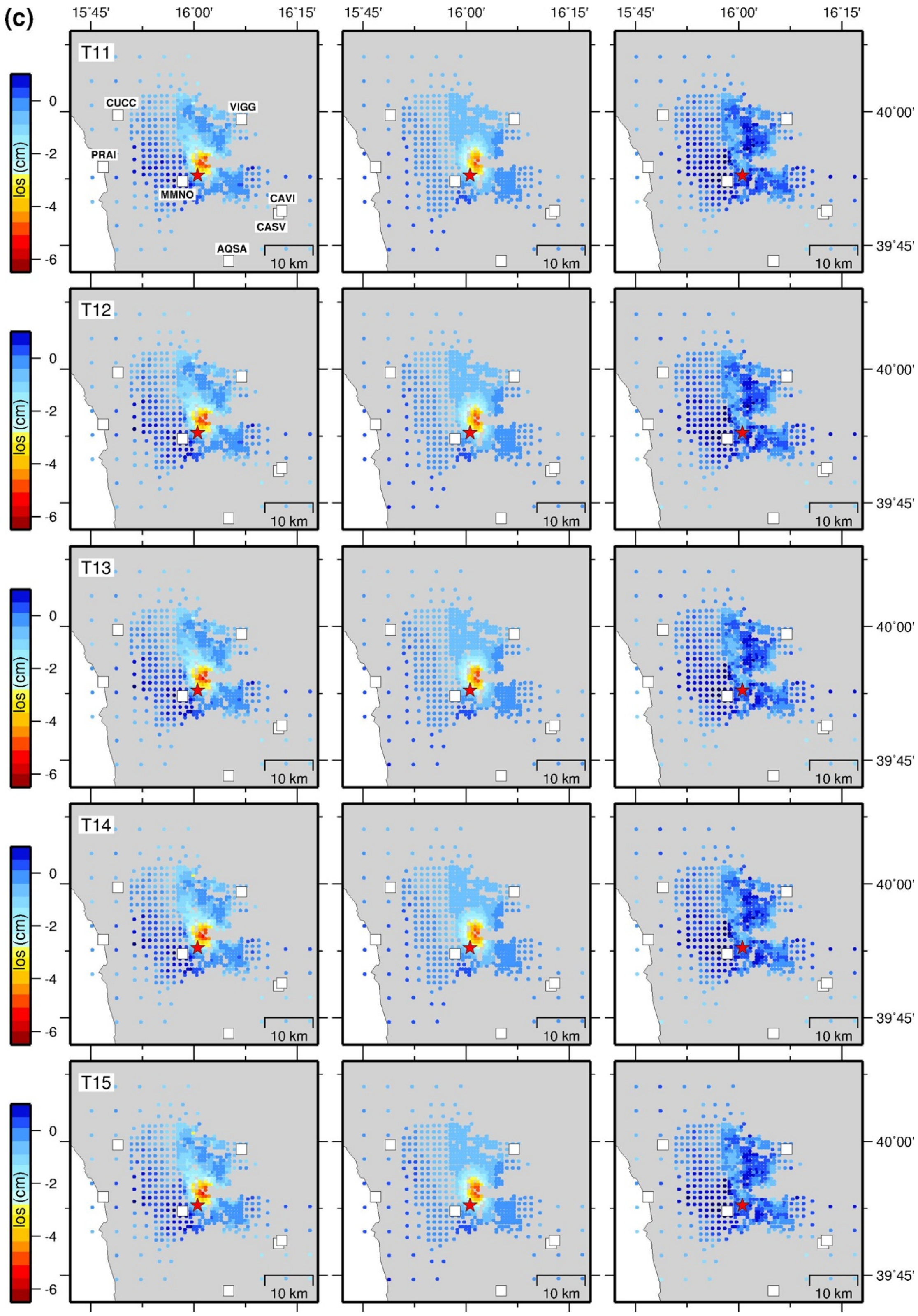

Supplementary Figure S7. (continued)

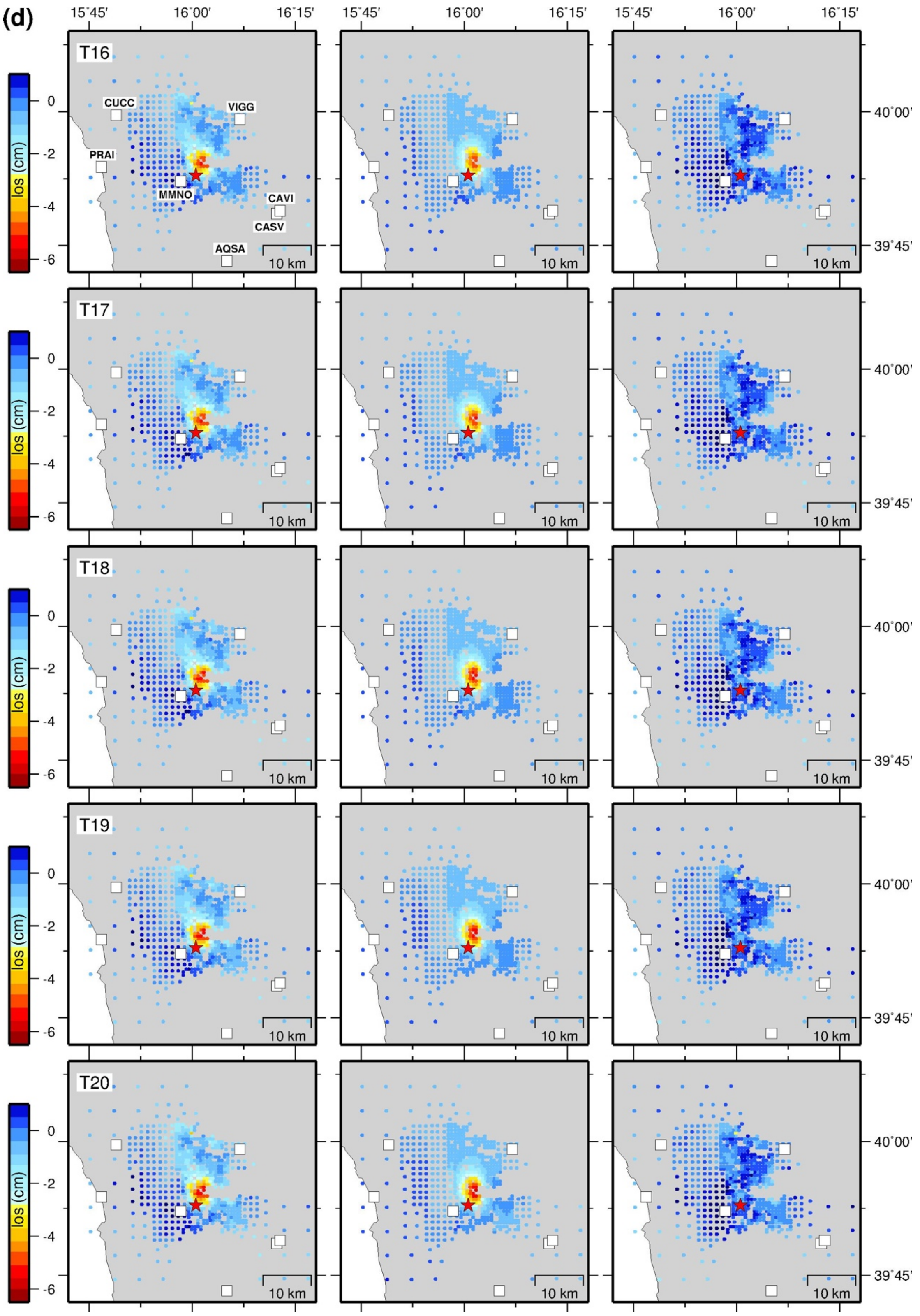

Supplementary Figure S7. (continued)

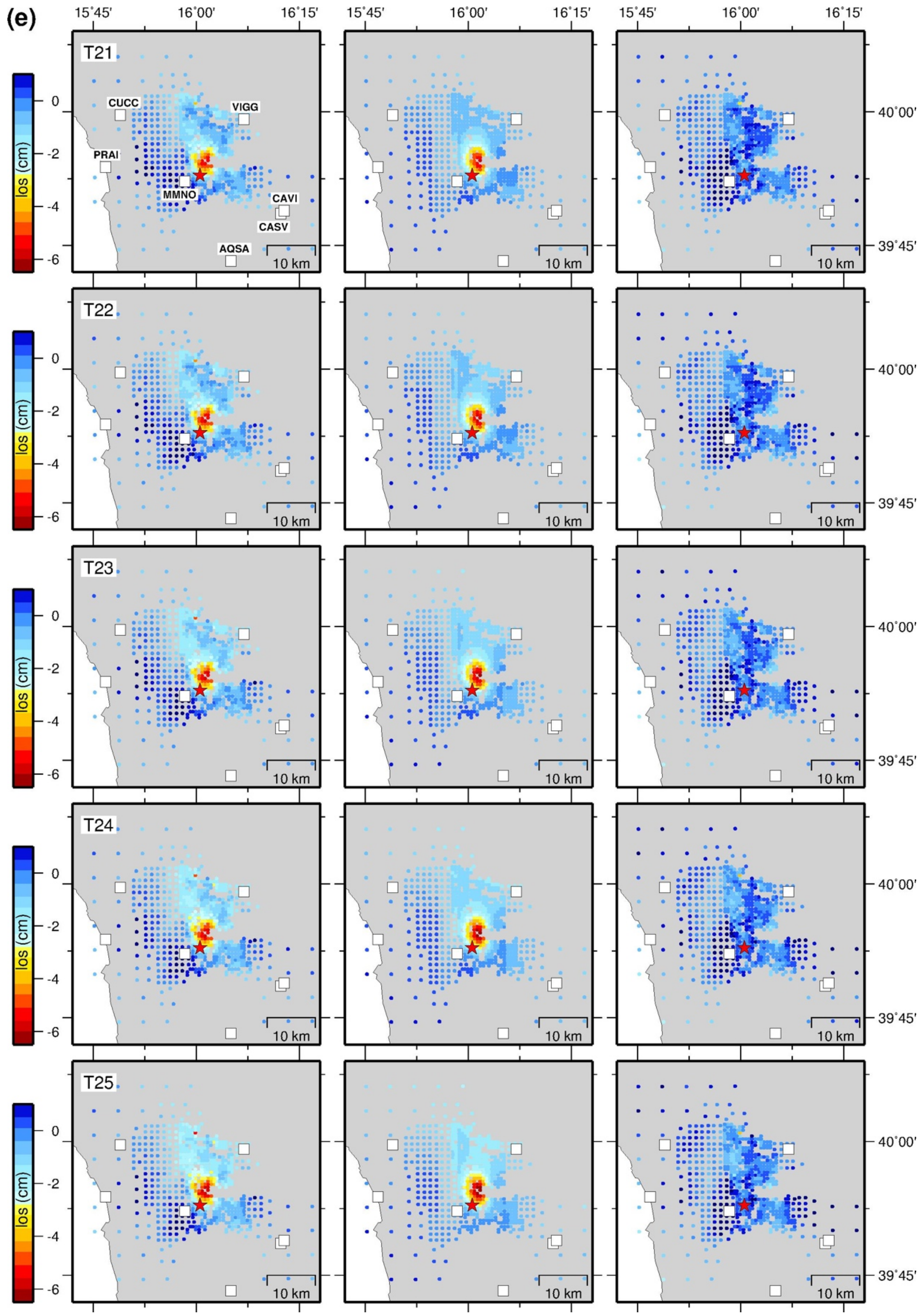

Supplementary Figure S7. (continued)

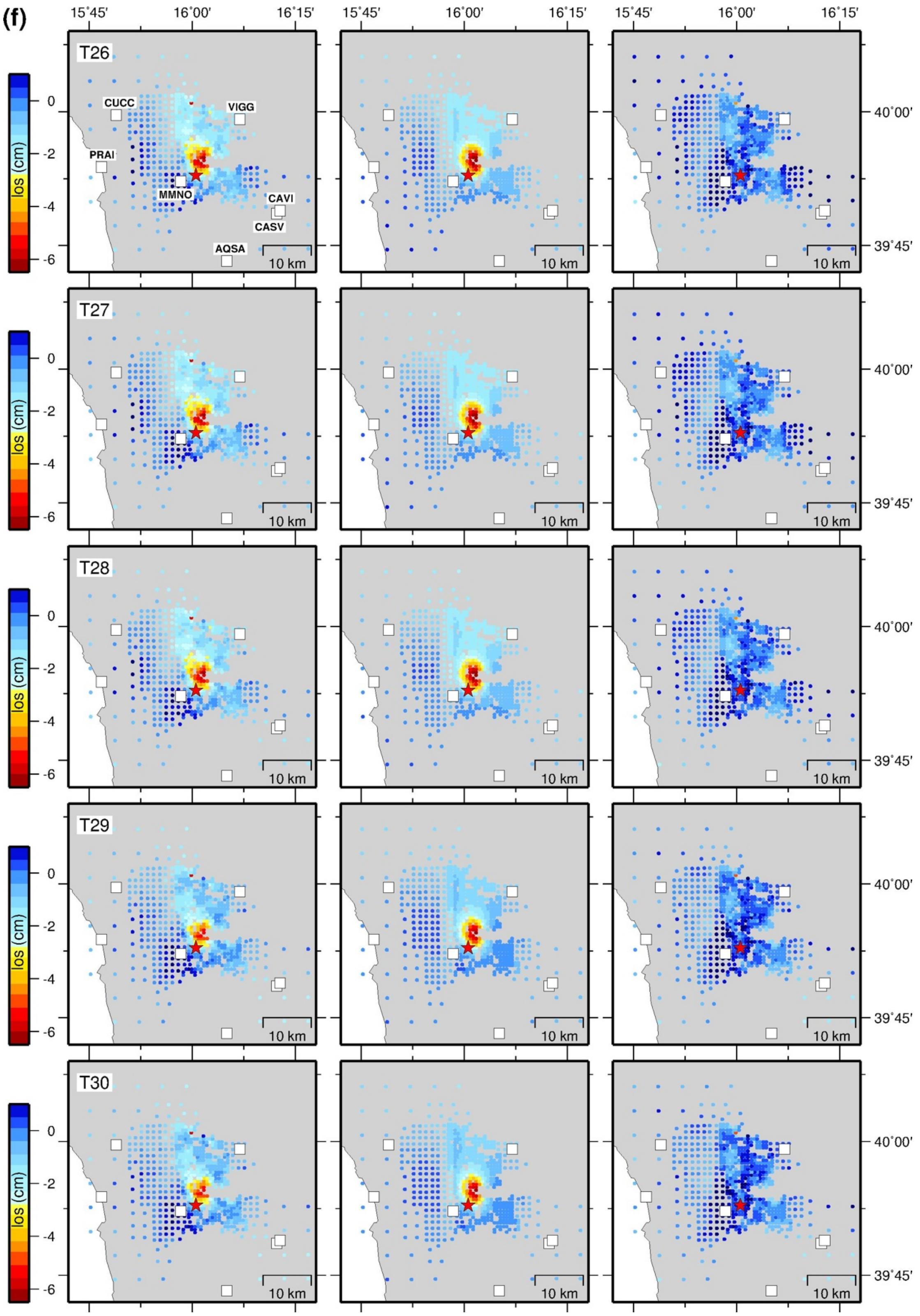

Supplementary Figure S7. (continued)

(g)

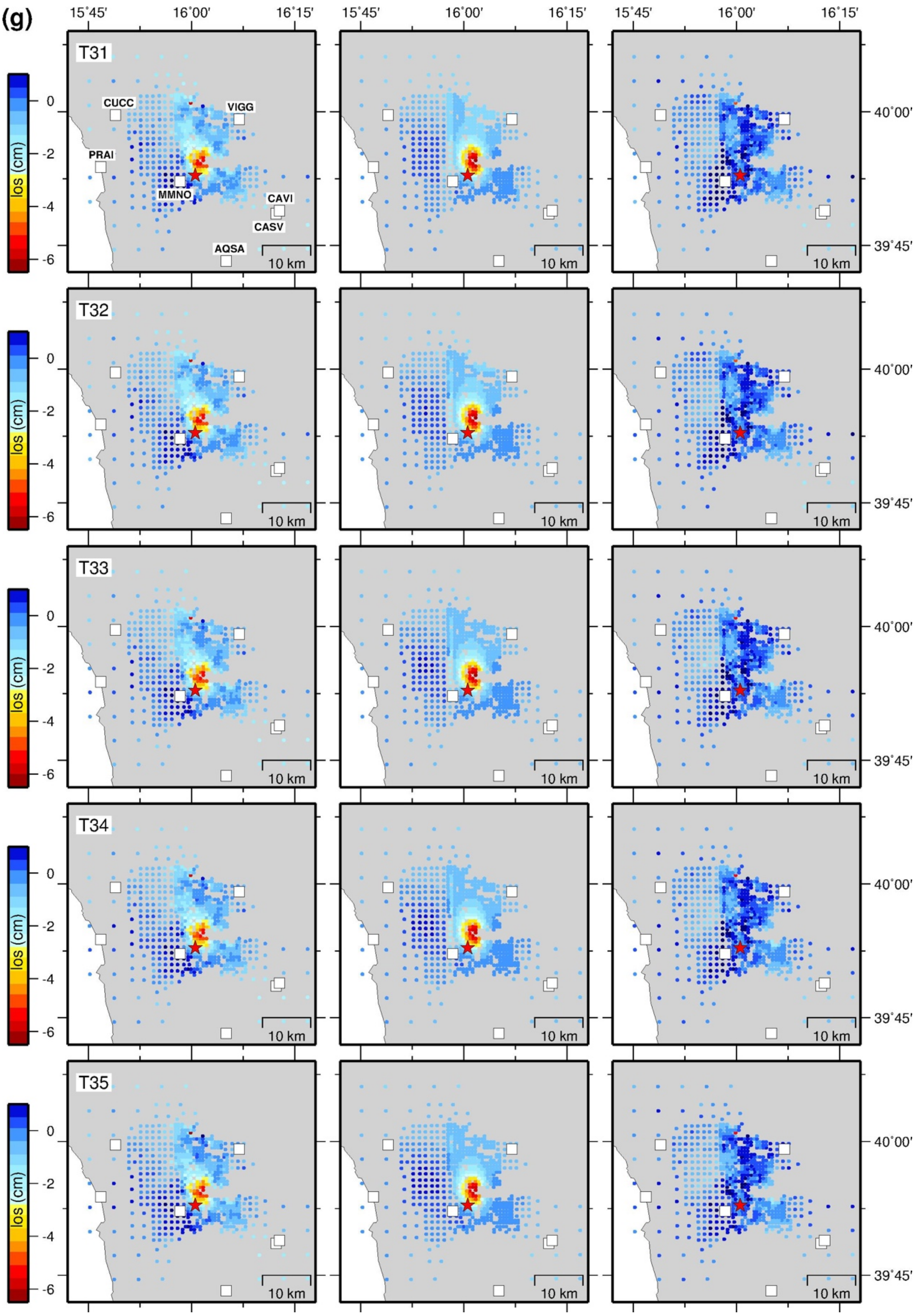

Supplementary Figure S7. (continued)

a) b)

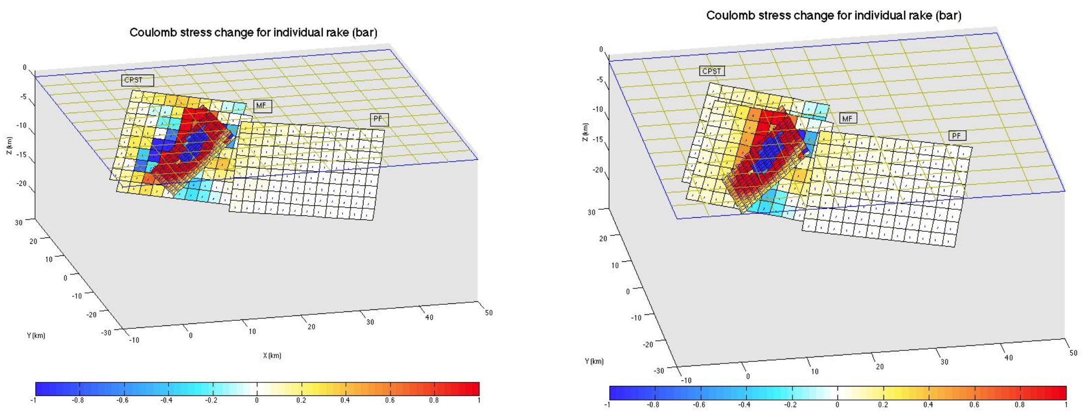

Supplementary Figure S8. Coulomb stress change resolved on faults surrounding the 2010-2014 Pollino swarm sequence due to the estimated mixed seismic/aseismic slip transient. (**a**) Change of Coulomb stress induced by the whole estimated aseismic slip; (**b**) change of Coulomb stress induced only by the main central patch of the estimated aseismic slip. The color range has been saturated at ±1 bar to highlight the stress changes on other faults (the maximum range is ±20 bar for patches on the fault of the modelled aseismic transient). MF, PF and CPST stand for Mercure, Pollino and Castelluccio Seluci-Timpa della Manca faults. The figure was created by using Coulomb software (Coulomb 3.1; https://earthquake.usgs.gov/research/software/coulomb/)[26].
